# A mutation in Nischarin causes otitis media via LIMK1 and NF-κB pathways

**DOI:** 10.1371/journal.pgen.1006969

**Published:** 2017-08-14

**Authors:** Michael Crompton, Tom Purnell, Hayley E. Tyrer, Andrew Parker, Greg Ball, Rachel E. Hardisty-Hughes, Richard Gale, Debbie Williams, Charlotte H. Dean, Michelle M. Simon, Ann-Marie Mallon, Sara Wells, Mahmood F. Bhutta, Martin J. Burton, Hilda Tateossian, Steve D. M. Brown

**Affiliations:** 1 Mammalian Genetics Unit, MRC Harwell Institute, Harwell, Oxfordshire, United Kingdom; 2 Nuffield Department of Surgical Sciences, University of Oxford, Oxford, Oxfordshire, United Kingdom; 3 Inflammation, Repair and Development Section, National Heart and Lung Institute, Imperial College London, London, United Kingdom; 4 Mary Lyon Centre, MRC Harwell Institute, Harwell, Oxfordshire, United Kingdom; 5 School of Paediatrics, University of Western Australia, Subiaco, Australia; Baylor College of Medicine, UNITED STATES

## Abstract

Otitis media (OM), inflammation of the middle ear (ME), is a common cause of conductive hearing impairment. Despite the importance of the disease, the aetiology of chronic and recurrent forms of middle ear inflammatory disease remains poorly understood. Studies of the human population suggest that there is a significant genetic component predisposing to the development of chronic OM, although the underlying genes are largely unknown. Using *N*-ethyl-*N*-nitrosourea mutagenesis we identified a recessive mouse mutant, *edison*, that spontaneously develops a conductive hearing loss due to chronic OM. The causal mutation was identified as a missense change, L972P, in the Nischarin (NISCH) gene. *edison* mice develop a serous or granulocytic effusion, increasingly macrophage and neutrophil rich with age, along with a thickened, inflamed mucoperiosteum. We also identified a second hypomorphic allele, V33A, with only modest increases in auditory thresholds and reduced incidence of OM. NISCH interacts with several proteins, including ITGA5 that is thought to have a role in modulating VEGF-induced angiogenesis and vascularization. We identified a significant genetic interaction between *Nisch* and *Itga5*; mice heterozygous for *Itga5*-null and homozygous for *edison* mutations display a significantly increased penetrance and severity of chronic OM. In order to understand the pathological mechanisms underlying the OM phenotype, we studied interacting partners to NISCH along with downstream signalling molecules in the middle ear epithelia of *edison* mouse. Our analysis implicates PAK1 and RAC1, and downstream signalling in LIMK1 and NF-κB pathways in the development of chronic OM.

## Author summary

Otitis media (OM) is the most common cause of deafness in children and is primarily characterised by inflammation of the middle ear. It is the most common cause of surgery in children in the developed world, with many children developing recurrent and chronic forms of OM undergoing tympanostomy tube insertion. There is evidence that a significant genetic component contributes towards the development of recurrent and chronic forms of OM. The mouse has been a powerful tool for identifying the genes involved in chronic OM. In this study we identified and characterised *edison*, a novel mouse model of chronic OM that shares important features with the chronic disease in humans. A mutation in the *Nisch* gene causes *edison* mice to spontaneously develop OM following birth and subsequently develop chronic OM, with an associated hearing loss. Our molecular analysis of the mutation reveals the underlying pathological mechanisms and pathways involved in OM in the *edison* mouse, involving PAK1, RAC1 and downstream signalling in LIMK1 and NF-κB pathways. Identification of the *edison* mutant provides an important genetic disease model of chronic OM and implicates a new gene and genetic pathways involved in predisposition to OM.

## Introduction

Otitis media (OM) is characterised by inflammation of the middle ear (ME), often associated with a conductive hearing impairment, and is the commonest cause of hearing loss in children. It is perceived by many to be a transient affliction that in reality places a substantial social, medical and economic burden on healthcare systems globally [1]. Evidence from studies of the human population suggests that there is a significant genetic component predisposing to the development of recurrent and chronic forms of OM [2,3]. Despite the importance of the disease, many of the genes involved in OM susceptibility have still yet to be identified. At present, the use of mouse models is the most promising method to identify candidate loci underlying susceptibility to OM. Mouse models have highlighted the role of Toll-like receptors (TLRs) in acute OM, in particular the protection against commensal and pathogenic bacteria, and that persistent NF-κB or TGF-β signalling could be two mechanisms leading to the overactive pro-inflammatory response seen in chronic OM [4,5].

The large-scale phenotype-driven mouse ENU (*N*-ethyl-*N*-nitrosourea) mutagenesis program at MRC Harwell [6,7] has previously identified two novel mouse mutants, *Jeff* and *Junbo*, that develop a conductive hearing loss characterised by ME fluid and mucosal inflammation [8,9]. The *Jeff* mouse has a mutation in the *Fbxo11* gene [10] and the *Junbo* mouse has a mutation in *Evi1* [9]. Studies have revealed that these genes, are involved in signalling of the TGF-β superfamily, via SMAD proteins [11,12]; negatively regulate NF-κB–dependent inflammation [13]; and highlight the role of HIF–VEGF pathways in the underlying genetic and pathophysiological mechanisms that predispose to chronic OM [14].

OM mouse models with single gene mutations have identified a number of genes as candidate susceptibility genes for human OM, including *Tlr2*, *Tlr4*, *p73*, *E2f4*, *Plg*, *Tgif1*, *Evi1* and *Fbxo11* [4]. These genes identified from mouse models of OM are beginning to be studied in the human population; with significant associations between OM and polymorphisms in *FBXO11* [15,16], *TLR2* [17] and *TLR4* [17–19].

We have identified and characterised a novel OM mouse mutant, *edison*, from the ENU mutagenesis program at MRC Harwell. Homozygous *edison* mice spontaneously develop a conductive hearing loss associated with chronic inflammation of the ME, sharing many features with chronic OM in humans. The underlying mutation of this phenotype has been identified as a mutation in the *Nisch* gene. We have explored the role of *Nisch* in chronic OM, relating the *edison* phenotype to the underlying mechanisms of *Nisch* function. We have utilised double mutants to assess genetic interactions and pathways involved, implicating PAK1 and RAC1, and downstream signalling events in LIMK1 and NF-κB signalling pathways in the development of chronic OM. Overall, the *edison* mouse highlights a new candidate gene for susceptibility to chronic OM and has provided further insight into the genetic pathways and pathogenic processes involved.

## Results

### Identification of the *edison* mutation

**Fig 1:**
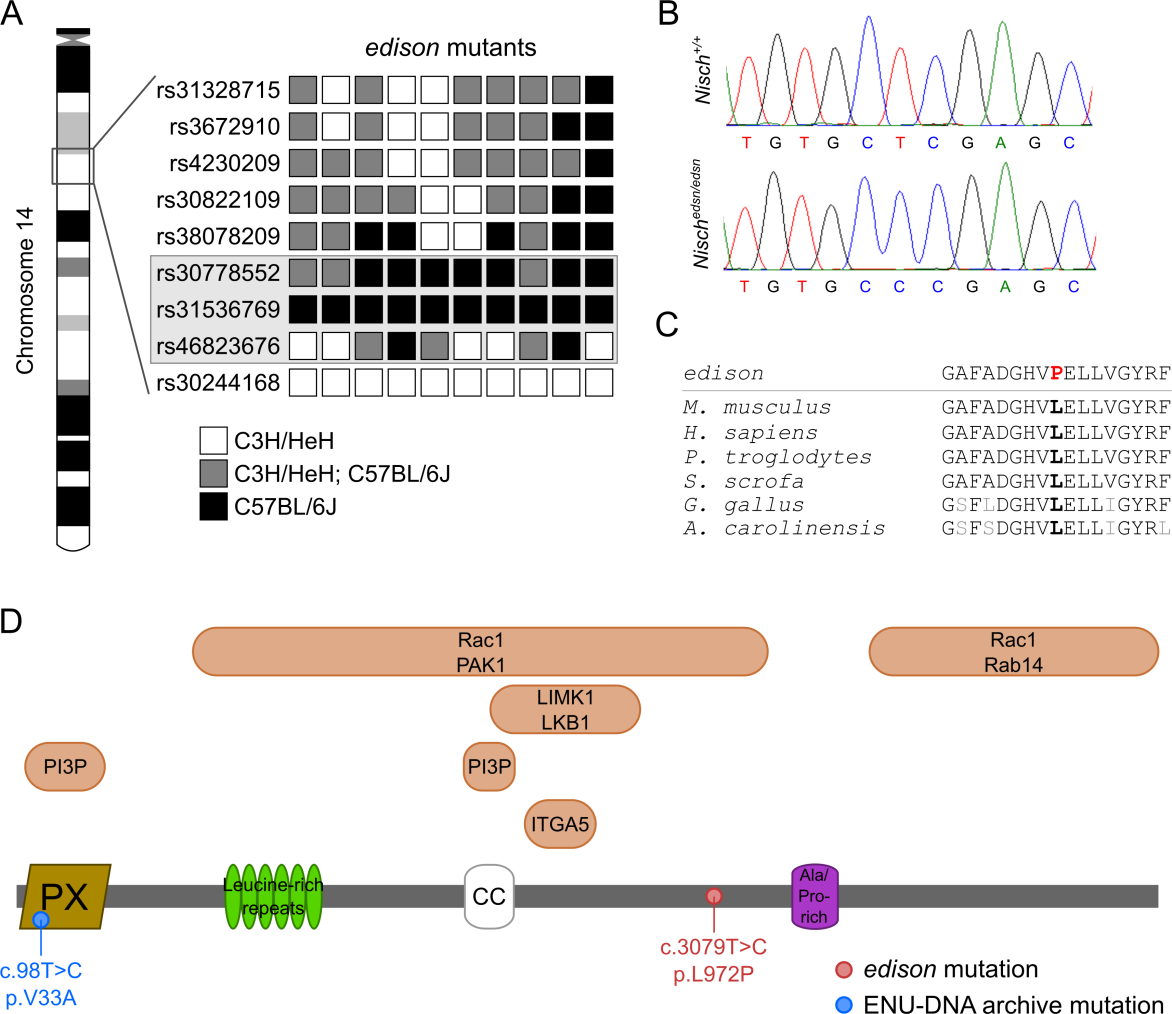
The *Nisch* gene is mutated in *edison* mice. **(A)** SNP mapping of 10 *edsn* mutants identified an approximately 9 Mb interval on chromosome 14 delineated by marker rs30778552 and rs46823676. The grayed box indicates the chromosomal interval bearing the *edsn* mutation. **(B)** Sequence analysis of the *Nisch* locus in *Nisch*^*+/+*^ and *Nisch*^*edsn/edsn*^ DNA. A c.3079T>C transition is detected in *Nisch*^*edsn/edsn*^ mutants that is not present in *Nisch*^*+/+*^ DNA. **(C)** Conservation of the mutated leucine residue across species. *Mus musculus*, ENSMUSG00000021910; *Homo sapiens*, ENSG00000010322; *Pan troglodytes*, ENSPTRG00000015001; *Sus scrofa*, ENSSSCG00000011442; *Gallus gallus*, ENSGALG00000043825; *Anolis carolinensis*, ENSACAG00000006939. **(D)** Schematic of the full length NISCH peptide (1593 amino acids). The molecule consists of a phox-homology (PX) domain, six leucine-rich repeats, a coiled-coil (CC) domain and an alanine/proline-rich region. Both the PX and CC domains of Nischarin are essential for endosomal targeting and interaction with phosphatidylinositol 3-phosphate (PI3P) in PI3P-enriched endosomes. Amino acids 709–807 of Nischarin interact with the integrin α5 (ITGA5) cytoplasmic tail. Both LIMK1 and LKB1 interact with positions 661–869 of Nischarin. Residues 246–1047 of Nischarin interact with PAK1. Rab14 interacts with amino acids 1190–1593. Finally, Rac1 interacts with two regions of Nischarin, amino acids 246–1047 and 1190–1593. The positions of the *Nisch*^*edsn*^ and *Nisch*^*V33A*^ mutations are also indicated.

A phenotype-driven ENU mutagenesis screen [20] identified a new recessive mutant, *edison* (*edsn*), with hearing loss. Preliminary phenotyping using a click-box test (20 kHz, 90 dB sound pressure level (SPL) tone burst) of an age-matched cohort derived from the founder mouse indicated that 6-week-old (wk) mice demonstrated a reduced startle response. SNP-based linkage analysis and mapping identified an approximately 9 Mb interval on chromosome 14 delineated by marker rs30778552 and rs46823676 containing 119 genes (Fig 1A). Whole-genome sequencing identified 60 ENU-induced, *de novo* variants within the critical 9Mb interval and importantly only one missense variant was identified. This missense variant was a c.3079T>C substitution in Nischarin (*Nisch*) (open reading frame of NCBI RefSeq transcript NM_022656.2) that results in a Leu972Pro substitution (Fig 1B). The change occurs in a highly conserved region that has been maintained through evolution (Fig 1C). PROVEAN analysis predicts that this change is ‘‘deleterious” and SIFT predicts that it is ‘‘not tolerated”. No other non-synonymous sequence changes were identified within the minimal interval. The *Nisch* locus encodes a protein of 1,593 amino acids, coded for by 22 exons. The protein consists of an N-terminal phox homology (PX) domain, six putative leucine-rich repeats (LRRs), a predicted coiled-coil (CC) domain, an alanine/proline-rich region and a long C-terminal region (Fig 1D).

DNA and sperm archives derived from ENU mutagenesis programmes [21] were utilised to identify an additional allele at the *Nisch* locus. We screened ten exons of *Nisch* employing high resolution melting analysis of ~10,000 mutant mice and identified a c.98T>C substitution resulting in a Val33Ala substitution within a conserved region of the NISCH PX domain. We rederived this second allele, *Nisch*^*V33A*^, and examined the phenotypes.

### Anatomical and histological analysis of *edison* mutant

#### Reduced numbers of *edison* progeny

Heterozygous animals (*Nisch*^*edsn/+*^) were intercrossed to generate wild-type control, heterozygous and homozygous (*Nisch*^*edsn/edsn*^) populations for the study and the progeny were genotyped. The homozygous mutants were viable, but the frequency of homozygous mutant offspring at weaning age was 15.9% (34 of 214), which was below the expected Mendelian ratio (χ^2^ test: *p* = 0.0012). To produce additional homozygous mice for this study, we crossed *Nisch*^*edsn/+*^ females with *Nisch*^*edsn/edsn*^ males. The frequency of surviving *Nisch*^*edsn/edsn*^ mice at weaning age was 33.0% (69 of 212), again less than the expected Mendelian ratio (χ^2^ test: *p* < 0.0001). Around 35% of the *Nisch*^*edsn/edsn*^ animals from both crosses were missing by weaning age, suggesting embryonic or neonatal lethality. Retarded growth was also observed in *edison* mice (S1A Fig). We compared weights of *Nisch*^*edsn/edsn*^ mice to wild-type littermates (S1B Fig) and found male *Nisch*^*edsn/edsn*^ mice to be 35% smaller (mean weight at 20 wk: *Nisch*^*+/+*^, 43.9 ± 0.72 g, n = 9; *Nisch*^*edsn/edsn*^, 27.2 ± 0.82 g, n = 18; Kruskall-Wallis: *p* < 0.001). Additionally, male *Nisch*^*edsn/+*^ mice were found to be 8% smaller than wild-type littermates (mean weight at 20 wk: *Nisch*^*edsn/+*^, 38.7 ± 0.57 g, n = 20; Kruskall-Wallis: *p* < 0.001). Similar observations were seen in female *Nisch*^*edsn/+*^ and *Nisch*^*edsn/edsn*^ mice compared to wild-type littermates (mean weight at 20 wk: *Nisch*^*+/+*^, 42.7 ± 1.02 g, n = 12; *Nisch*^*edsn/+*^, 40.2 ± 0.67 g, n = 17; *Nisch*^*edsn/edsn*^, 26.6 ± 1.09 g, n = 13; Kruskall-Wallis: *Nisch*^*edsn/+*^, *p* = 0.057; *Nisch*^*edsn/edsn*^, *p* < 0.001).

#### Mild craniofacial defects in *edison* mice

We found that all *Nisch*^*edsn/edsn*^ mice demonstrated a shortened snout compared with wild-type mice, indicating a mild craniofacial abnormality (S1 Fig). To investigate the craniofacial phenotype of mice, we took dorsoventral X-ray images of the skulls and computed cranial measurements (S1C Fig). To determine whether any of the cranial bones showed disproportionate growth, allometric comparisons against skull length were used to normalise the data for differences in body size (S1C and S1D Fig). Evaluation of *Nisch*^*edsn/edsn*^ and wild-type skulls revealed a significant difference in allometric comparisons between nasal bone/skull length (Kruskal- Wallis, *p* < 0.001) and frontal bone/skull length (Kruskall-Wallis: *p* = 0.036). The magnitude of the difference between *Nisch*^*edsn/edsn*^ and wild-type mice was small (~3%). No other skeletal defects were evident.

To investigate the orientation and morphology of the Eustachian tube (ET) in *edison* mice, the skulls were stained and the angles between the midline of the skull and the bony part of the left and right ET were measured (S1E and S1F Fig). No statistically significant differences in ET measurements were detected between *Nisch*^*edsn/edsn*^ and wild-type mice (Kruskall-Wallis: Left, *p* = 0.848; Right, *p* = 0.324).

#### Hearing deficits in *Nisch*^*edsn/edsn*^ animals due to spontaneous otitis media

**Fig 2:**
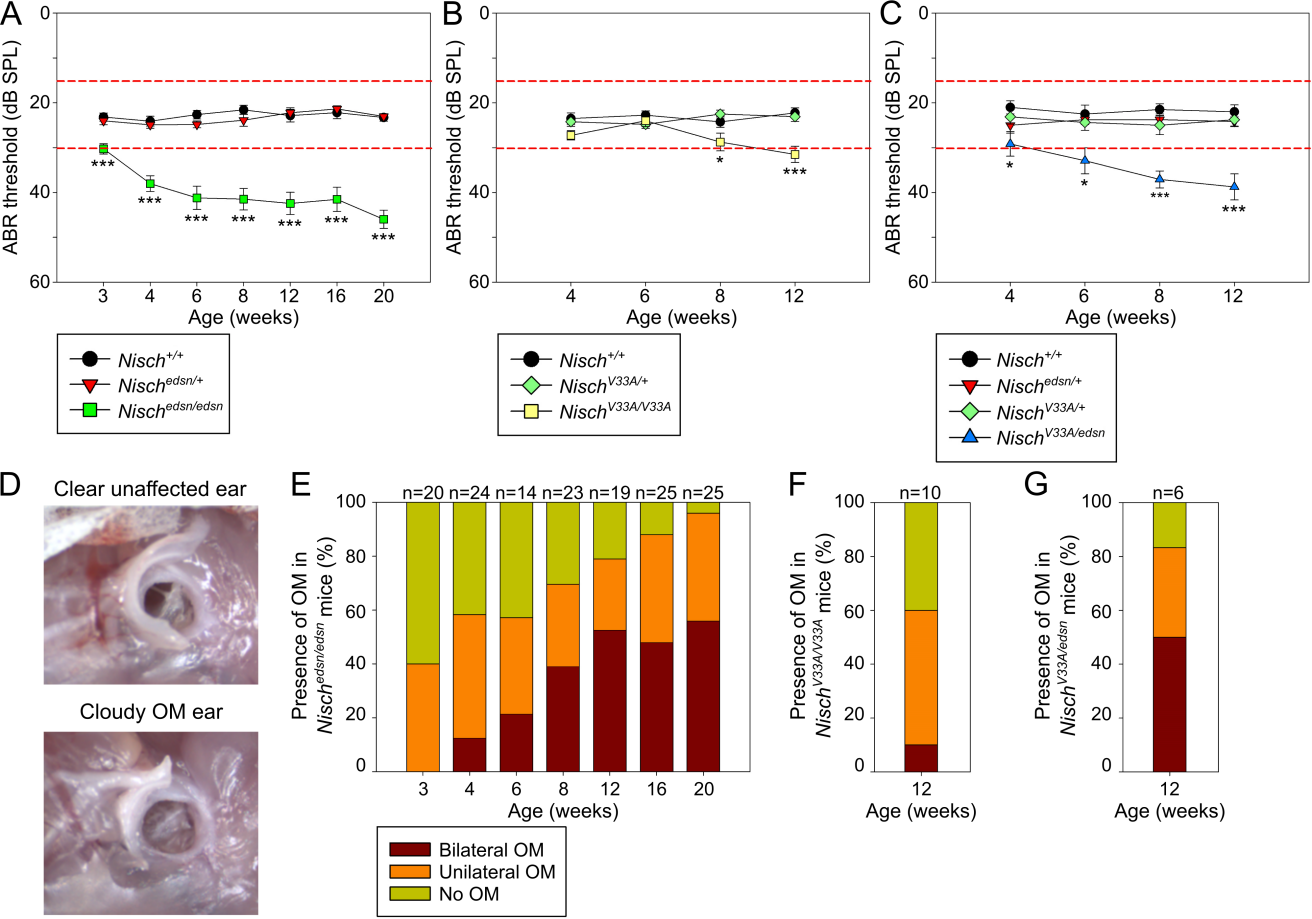
Mild to moderate hearing impairments in *Nisch*^*edsn/edsn*^, *Nisch*^*V33A/V33A*^ and *Nisch*^*edsn/V33A*^ mice due to otitis media. **(A-C)** Click-evoked ABR thresholds across a time course show a mild to moderate progressive hearing impairment in (A) *Nisch*^*edsn/edsn*^, (B) *Nisch*^*V33A/V33A*^ and (C) *Nisch*^*V33A/edsn*^ mice. Expected ABR threshold range for normal hearing was between 15–30 dB SPL (dashed red lines). * *P* < 0.05; *** *P* < 0.001. (A) *Nisch*^*+/+*^, n = 10–33; *Nisch*^*edsn/+*^, n = 12–33; *Nisch*^*edsn/edsn*^, n = 14–25. (B) *Nisch*^*+/+*^, n = 10; *Nisch*^*V33A/+*^, n = 13; *Nisch*^*V33A/V33A*^, n = 10. (C) *Nisch*^*+/+*^, n = 5; *Nisch*^*edsn/+*^, n = 6; *Nisch*^*V33A/+*^, n = 4; *Nisch*^*V33A/edsn*^, n = 6. Error bars indicate standard error of mean. A Kruskall-Wallis test was performed followed by Dunn’s multiple comparison tests for post-hoc analysis. **(D)** Visual inspection of the tympanic membrane is a simple method for diagnosing OM. Wild-type (*Nisch*^*+/+*^) mice have no visible fluid behind the tympanic membrane and the malleus is easily recognizable, while affected *Nisch*^*edsn/edsn*^ mice have fluid behind the tympanic membrane and the malleus is obscured. **(E)** The incidence of bilateral and unilateral OM increases in prevalence with age in *Nisch*^*edsn/edsn*^ mice. **(F, G)** At 12 wk, an increased proportion of (F) *Nisch*^*V33A/V33A*^ and (G) *Nisch*^*V33A/edsn*^ mice display bilateral and unilateral OM.

In order to assess the onset and progression of the hearing impairment in *edison* mice, click-evoked ABR tests to measure auditory function were performed in cohorts of age matched littermates from 3 wk to 20 wk (Fig 2A). *Nisch*^*+/+*^ and *Nisch*^*edsn/+*^ mice showed normal ABR thresholds throughout the time course, whereas significantly elevated thresholds were detected in *Nisch*^*edsn/edsn*^ mice as early as 3 wk and progressively increased with age (mean ABR threshold: *Nisch*^*edsn/edsn*^, 3 wk = 30 ± 1 dB SPL, n = 36; 20 wk = 46 ± 2 dB SPL, n = 61; Kruskall-Wallis: *p* < 0.001). We showed that the mean ABR thresholds were elevated by about 20–30 dB SPL in *Nisch*^*edsn/edsn*^ animals when compared with wild-type mice, suggesting a conductive hearing loss.

Visual inspection of the tympanic membrane is a simple method for diagnosing OM. The cloudy appearance of an eardrum is a semi-quantitative measure of bulla fluid accumulation and the incidence of OM was assessed in *edison* mice (Fig 2D). *Nisch*^*+/+*^ and *Nisch*^*edsn/+*^ animals showed a small incidence of unilateral OM (*Nisch*^*+/+*^, 1% unilateral OM, n = 148; *Nisch*^*edsn/+*^, 2% unilateral OM, n = 194). In *Nisch*^*edsn/edsn*^ animals there was a clear trend showing increased prevalence of OM from 3 wk onwards (Fig 2E). At 3 wk, no *Nisch*^*edsn/edsn*^ mice had bilateral OM, 40% had unilateral OM and 60% had no OM phenotype (Fisher Exact: *p* = 0.028). The prevalence of OM progressively increased throughout the time course, where at 20 wk 56% of *Nisch*^*edsn/edsn*^ mice had bilateral OM, 40% had unilateral OM and only 4% displayed no OM phenotype (Fisher Exact: *p* < 0.001).

In *Nisch*^*V33A/V33A*^ animals, examination of auditory function revealed that mice develop a milder hearing deficit compared to *Nisch*^*edsn/edsn*^ mice (Fig 2B). Although progressive, the onset of the hearing deficit was observed at 8 wk in *Nisch*^*V33A/V33A*^ animals. Visual inspection of the tympanic membrane was used to assess the incidence of OM in *Nisch*^*V33A/V33A*^ mice (Fig 2F). At 12 wk, *Nisch*^*V33A/V33A*^ mice exhibited significantly increased prevalence of OM compared to wild-type littermates (Fisher Exact: *p* = 0.011). In *Nisch*^*V33A/V33A*^ mutants, 10% had bilateral OM, 50% had unilateral OM and only 40% displayed no OM phenotype (n = 10). The less significant hearing deficit and limited disease progression suggests that the *Nisch*^*V33A*^ allele is hypomorphic in nature. Compound heterozygotes carrying both *Nisch*^*edsn*^ and *Nisch*^*V33A*^ alleles showed a similar phenotype to *Nisch*^*edsn/edsn*^ mice (Fig 2C and 2G). *Nisch*^*V33A/edsn*^ mice develop a spontaneous chronic OM, with an associated elevation in ABR hearing thresholds. The hearing deficit was progressive, with onset recorded at 4 wk in *Nisch*^*V33A/edsn*^ mice. At 12 wk, the incidence of OM in *Nisch*^*V33A/edsn*^ mice was assessed and 50% had bilateral OM, 33% had unilateral OM and only 17% displayed no OM phenotype (n = 6) (Fig 2G). This increased prevalence of OM in *Nisch*^*V33A/edsn*^ mice at 12 wk was significantly different compared to wild-type littermates (Fisher Exact: *p* = 0.015).

We assessed whether there was a sensorineural element to the hearing loss in *Nisch*^*edsn/edsn*^ mice by evaluating frequency-specific auditory function and the inner ear morphology (S2 Fig). We tested 5 mice for each genotype from 4 wk to 20 wk for ABR response at 3 frequencies 8, 16, and 32 kHz. Parallel shifts in audiometric profiles across frequencies at the different ages were recorded, consistent with an underlying conductive hearing loss (S2A Fig). We analysed the inner ear morphology from 5 mice for each genotype at 20 wk to examine the structure of the organ of Corti and the sensory cells (S2B–S2D Fig). Histological examination of mid-modiolar sections of the cochlea revealed no obvious abnormalities in *Nisch*^*edsn/edsn*^ mice. Similarly, ultra-structural analysis of the inner ear with scanning electron microscopy showed no evidence of hair cell damage or hair cell loss, with *Nisch*^*edsn/edsn*^ mice displaying normal cell and bundle morphology throughout the cochlear turn (basal, mid and apical).

#### Histological analysis of middle ear demonstrates chronic otitis media

**Fig 3:**
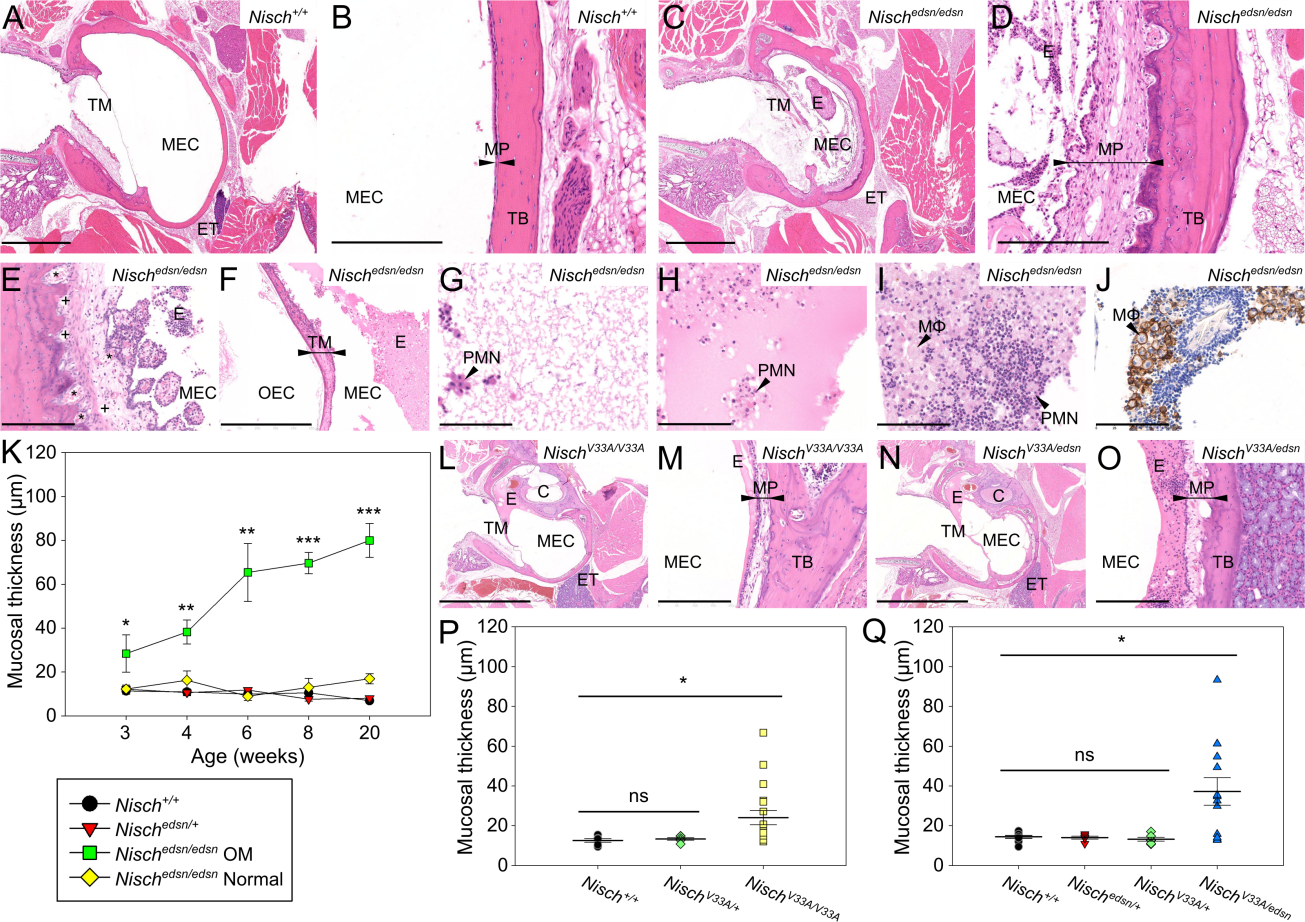
Middle ear histology indicates chronic otitis media in *Nisch*^*edsn/edsn*^ mice. **(A-D)** H&E stained transverse sections of the MEC and mucoperiosteum, in 20 wk (A, B) *Nisch*^*+/+*^ and (C, D) *Nisch*^*edsn/edsn*^ mice. Wild-type animals have no inflammation or exudate in the MEC and a thin mucoperiosteum covers the temporal bone. *Nisch*^*edsn/edsn*^ mice exhibit chronic inflammation with an exudate and thickened mucoperiosteum. **(E, F)** Additional characteristics of chronic OM in *Nisch*^*edsn/edsn*^ middle ears; changes include (E) fibrous polyps, with dilated lymphatic (+) and blood vessels (*) in the mucoperiosteum, and (F) fibrous thickening of the tympanic membrane. **(G-I)** Differing types of middle ear effusion; from (G) a thin watery effusion, (I) a dense granulocyte-rich effusion, or (H) a combination of both. **(J)** Immunohistochemical staining of a *Nisch*^*edsn/edsn*^ ear with OM using an F4/80 antibody shows a cellular exudate rich in macrophages (brown). Representative image from four mice **(K)** Blinded assessment of mean mucosal thickness displays a progressive thickening in *Nisch*^*edsn/edsn*^ ears with OM, showing significant increases throughout the study. There is no histological difference seen in mean mucosal thickness between *Nisch*^*edsn/edsn*^ Normal ears and wild-type littermates. The criteria used to impartially categorise the ears into *Nisch*^*edsn/edsn*^ OM and *Nisch*^*edsn/edsn*^ Normal was from visualisation of tympanic membrane. *Nisch*^*+/+*^ n = 10–14; *Nisch*^*edsn/+*^ n = 10–22; *Nisch*^*edsn/edsn*^ OM n = 8–16; *Nisch*^*edsn/edsn*^ Normal n = 3–10. **(L, M, N, O)** H&E stained transverse sections of the MEC and mucoperiosteum, in 12 wk (L, M) *Nisch*^*V33A/V33A*^ and (N, O) *Nisch*^*V33A/edsn*^ mice, displaying chronic OM in both. **(P, Q)** Blinded assessment of mean mucosal thickness shows significant increases in both (P) *Nisch*^*V33A/V33A*^ and (Q) *Nisch*^*V33A/edsn*^ mice. Combined data is presented including mice with OM and mice with clear ears. (P) *Nisch*^*+/+*^ n = 6; *Nisch*^*V33A/+*^ n = 6; *Nisch*^*V33A/V33A*^ n = 18. (Q) *Nisch*^*+/+*^ n = 10; *Nisch*^*edsn/+*^ n = 6; *Nisch*^*V33A/+*^ n = 6; *Nisch*^*V33A/edsn*^ n = 12. C, cochlea; ET, eustachian tube; E, exudate; MΦ, foamy macrophage; MEC, middle ear cavity; MP, mucoperiosteum (arrowheads); PMN, polymorphonuclear cells; TB, temporal bone; TM, tympanic membrane; *, blood vessels; +, lymphatic vessels. A, C, L, N scale bar = 2 mm; F scale bar = 500 μm; B, D, E, M, O scale bar = 200 μm; G-J scale bar = 100 μm. * *P* < 0.05; ** *P* < 0.01; *** *P* < 0.001. Error bars indicate standard error of mean. A Kruskall-Wallis test was performed followed by Dunn’s multiple comparison tests for post-hoc analysis.

Histological examination was performed to investigate the pathological changes in the ME of *edison* mice (Fig 3). Wild-type and *Nisch*^*edsn/+*^ mice at 20 weeks displayed clear ME cavities, lined with a thin mucoperiosteum (Fig 3A and 3B). *Nisch*^*edsn/edsn*^ mice develop chronic otitis media with ME cavities filled with a cellular exudate and lined with a thickened mucoperiosteum (Fig 3C and 3D). The mucosal inflammation was diffuse and of moderate severity. Papillary to polypoid exophytic growths were observed projecting into the ME cavity (Fig 3E). The polypoid projections were most likely due to fibroblast stimulation as a consequence of chronic inflammation. There were small accumulations of inflammatory cells and dilated lymphatic and blood vessels in the mucoperiosteum (Fig 3E). In middle ears with the most severe phenotype, there were instances showing inflammation of the tympanic membrane (Fig 3F). There were differing types of ME effusion: a thin watery effusion, a dense granulocyte-rich effusion, or a combination of both (Fig 3G–3I). F4/80 stained sections identified increased macrophage infiltration in the exudates of some *Nisch*^*edsn/edsn*^ mice, with the presence of enlarged foamy macrophages containing coarsely granular material (Fig 3J).

To further investigate the ME phenotype in *edison*, heads were sectioned at 3, 4, 6, 8 and 20 wk. At 3 wk, most *Nisch*^*edsn/edsn*^ animals displayed a normal wild-type phenotype with clear ears (71%, n = 14 ears), and only three mice had a thin watery effusion (21%, n = 14 ears). At 4 wk, there were five *Nisch*^*edsn/edsn*^ ears that contained fluid in the ME cavity with a thickened epithelial lining (50%, n = 10 ears). Two ears had a thin watery effusion, and three ears displayed a cellular effusion with the presence of polymorphonuclear cells (PMNs) that are the first responding inflammatory cells that migrate towards the site of inflammation. In 6 wk *Nisch*^*edsn/edsn*^ animals, four ears showed inflammation in the ME cavity with a thickened epithelial lining (33%, n = 12 ears), with three of these ears also containing a cellular effusion. By 8 wk, the inflammation in *Nisch*^*edsn/edsn*^ animals had progressed to a chronic inflammation with effusion, as evidenced by a thick cellular fluid in the bulla cavity along with a greatly thickened ME mucosa (67%, n = 12 ears). There was variability in the type of effusion found, where two ears exhibited a serous effusion and in six ears the ME fluid was thick with the presence of macrophages and PMNs. *Nisch*^*edsn/edsn*^ mice studied at 20 wk displayed a chronic inflammation with effusion, similar to what was observed at 8 wk. Seventeen ears presented a chronic inflammation (77%, n = 22 ears), eleven of which contained fluid. Ten ears exhibited a thick effusion with the presence of macrophages and PMNs and in only one ear a serous exudate was observed. Cellular debris-like aggregates were present in the thick effusions and developing polyps were observed in the ME mucosa. None of the wild-type or *Nisch*^*edsn/+*^ mice evaluated displayed signs of OM pathology at any time point.

To quantify the chronic inflammation observed in *Nisch*^*edsn/edsn*^ mice and assess any subclinical changes in pathology, blinded assessment of the mucoperiosteum thickness was carried out in all *edison* genotypes from 3 wk to 20 wk (Fig 3K). As there was variation in the penetrance of OM in *Nisch*^*edsn/edsn*^ mice, the ears were categorised into those that had an OM phenotype and those that did not, *Nisch*^*edsn/edsn*^ OM and *Nisch*^*edsn/edsn*^ Normal respectively. The criteria used to impartially categorise the ears into *Nisch*^*edsn/edsn*^ OM and *Nisch*^*edsn/edsn*^ Normal was visualisation of tympanic membrane. *Nisch*^*edsn/edsn*^ Normal ears displayed normal, wild-type ME mucosal morphology of between 9.0 μm to 17.0 μm mucosal thickness, with no significant differences compared to wild-type littermates. In contrast, mucosal thickness observed in *Nisch*^*edsn/edsn*^ OM ears showed a mucosal morphology that progressively thickened with age. At 3 wk, *Nisch*^*edsn/edsn*^ OM ears had a mean mucosal thickness of 28.4 ± 8.49 μm and by 20 wk, the mean mucosal thickness had increased to 80.0 ± 7.73 μm. *Nisch*^*edsn/edsn*^ OM ears showed significantly increased thickening of the ME mucosa at every time point, when compared to *Nisch*^*+/+*^ ears (Kruskall-Wallis: 3 wk, *p* = 0.013; 4 wk, *p* = 0.001; 6 wk, *p* = 0.06; 8 wk, *p* < 0.001; 20 wk, *p* < 0.001).

Furthermore, histological analysis confirmed that *Nisch*^*V33A/V33A*^ and *Nisch*^*V33A/edsn*^ ears develop chronic OM with a fluid-filled cavity, lined with thickened mucoperiosteum (Fig 3L–3Q). However, there is a clear phenotypic gradient in the severity of OM seen in *Nisch*^*V33A/V33A*^, *Nisch*^*V33A/edsn*^ and *Nisch*^*edsn/edsn*^ mice. At 12 wk, *Nisch*^*V33A/V33A*^ mice had a mean mucosal thickness of 24.0 ± 3.60 μm (Kruskall-Wallis: *p* = 0.018) and *Nisch*^*V33A/edsn*^ mice had a mean mucosal thickness of 37.2 ± 6.98 μm (Kruskall-Wallis: *p* = 0.017).

#### Upregulation of hypoxia and inflammatory genes in middle ear fluid

Venous blood and ear fluid was used to perform RT-qPCR to look for expression changes in inflammatory and hypoxia responsive genes (S3 Fig). Relative to a normoxic baseline control of *Nisch*^*edsn/edsn*^ white blood cells (WBC), the inflammatory cells that accumulate within the bulla fluids of *Nisch*^*edsn/edsn*^ mice showed elevated expression of *Hif1a* (30-fold; t-test: *p* < 0.001) and the HIF responsive gene *Vegfa* (87-fold; t-test: *p* < 0.001). *Il1b* (15-fold; t-test: *p* = 0.003) and *Tnfa* (66-fold; t-test: *p* < 0.001) are known modulators of *Hif1a* translation and expression data indicated that they were also elevated in *Nisch*^*edsn/edsn*^ bulla fluid inflammatory cells relative to WBC (S3 Fig). Additionally expression of *Src*, which is a known mediator of VEGF signalling, was also significantly elevated in *Nisch*^*edsn/edsn*^ bulla compared to WBC (24-fold; t-test: *p* = 0.005). Finally, *Evi1* and *Fbxo11* were expressed in the bulla fluid of *Nisch*^*edsn/edsn*^ mice, but only *Evi1* (256-fold; t-test: *p* = 0.006) was expressed at higher levels relative to WBC.

#### Lung abnormalities

**Fig 4:**
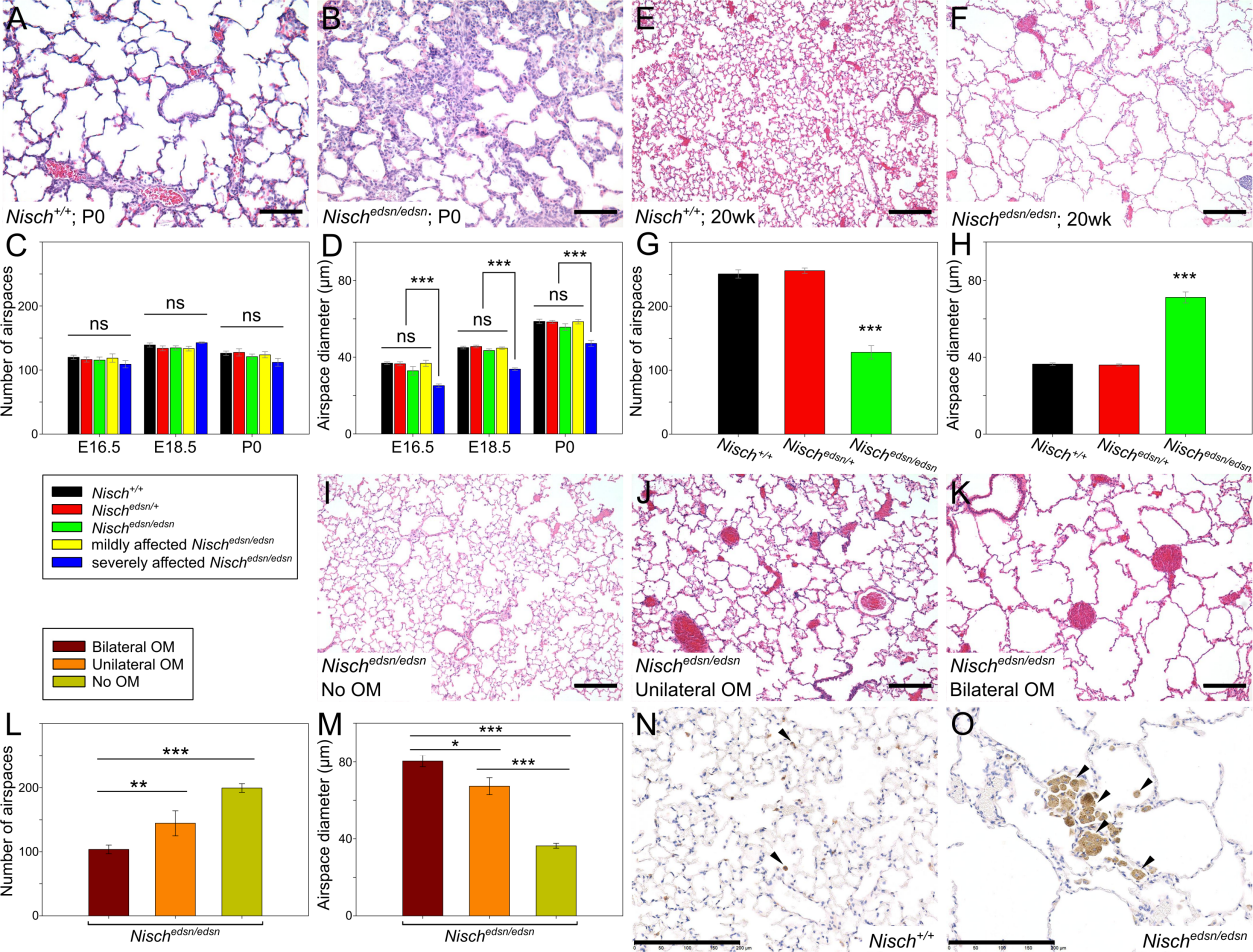
*Nisch*^*edsn/edsn*^ mice display embryonic and adult lung abnormalities. **(A-D)** At E16.5, E18.5 and P0, some *Nisch*^*edsn/edsn*^ embryos display lung abnormalities. H&E stained lung sections from (A) *Nisch*^*+/+*^ and (B) severely affected *Nisch*^*edsn/edsn*^ mice at P0, show thickened interstitial mesenchyme and smaller airspaces in *Nisch*^*edsn/edsn*^ lungs. (C) Number of airspaces was counted in three different 6.5 x 10^5^ μm^2^ regions for all embryos and new born mice, with no significant difference between genotypes. (D) Airspace diameters were measured for 30 airspaces in three different regions for all embryos and new born mice. The mean airspace width in severely affected *Nisch*^*edsn/edsn*^ animals was significantly smaller. *Nisch*^*+/+*^ n = 6–9; *Nisch*^*edsn/+*^ n = 11–15; *Nisch*^*edsn/edsn*^ n = 6–10; mildly affected *Nisch*^*edsn/edsn*^ n = 4–7; severely affected *Nisch*^*edsn/edsn*^ n = 2–3. **(E-H)** Adult *Nisch*^*edsn/edsn*^ mice exhibit an emphysema-like phenotype. H&E stained lung sections from (E) *Nisch*^*+/+*^ and (F) *Nisch*^*edsn/edsn*^ animals at 20 wk, show enlargement of air spaces in *Nisch*^*edsn/edsn*^ lungs accompanied by disruption of normal alveolar architecture. (G) In *Nisch*^*edsn/edsn*^ lungs quantification of the number of airspaces indicated a significant decrease compared to wild-type and (H) the mean airspace width in *Nisch*^*edsn/edsn*^ lungs was significantly larger than in wild-type tissue. *Nisch*^*+/+*^ n = 10; *Nisch*^*edsn/+*^ n = 10; *Nisch*^*edsn/edsn*^ n = 18. **(I-M)** The severity of the emphysema-like lung phenotype observed in *Nisch*^*edsn/edsn*^ replicates the severity of the OM phenotype in the middle ear. H&E stained lung sections from *Nisch*^*edsn/edsn*^ animals at 20 wk show the increasing severity of the emphysema-like phenotype from mice with (I) no OM phenotype, to (J) unilateral OM and to (K) bilateral OM. (L) The mean number of airspaces in *Nisch*^*edsn/edsn*^ animals with no OM phenotype was significantly increased compared to *Nisch*^*edsn/edsn*^ animals with bilateral OM. (M) The mean airspace width in *Nisch*^*edsn/edsn*^ mice with no OM phenotype was significantly smaller than *Nisch*^*edsn/edsn*^ animals with bilateral OM. Bilateral OM n = 10; Unilateral OM n = 6; Clear n = 2. **(N, O)** Immunohistochemical staining of lung sections using an F4/80 antibody. Lung sections from (N) *Nisch*^*+/+*^ and (O) *Nisch*^*edsn/edsn*^ animals at 20 wk stained with F4/80 show collections of enlarged alveolar macrophages (brown) in *Nisch*^*edsn/edsn*^ lungs compared to wild-type littermates. Representative images from four mice per genotype. N, O scale bar = 200 μm; A, B, E, F, I, J, K scale bar = 100 μm. ns *P* > 0.05; * *P* < 0.05; ** *P* < 0.01; *** *P* < 0.001. Error bars indicate standard error of mean. Embryonic lung data in panels C and D were analysed by one-way ANOVAs and Holm-Sidak’s multiple comparison procedures for post-hoc testing. Adult lung data (G, H, L, M) was not normally distributed and a Kruskall-Wallis test was performed followed by Dunn’s multiple comparison tests for post-hoc analysis.

Due to the reduced numbers of *Nisch*^*edsn/edsn*^ progeny at weaning age, we studied the mice at E16.5, E18.5 and P0 to search for any physiological defect that may contribute to lethality (Fig 4A–4D). In some *Nisch*^*edsn/edsn*^ mice we observed morphological lung defects including thickened interstitial mesenchyme and smaller airspaces than in wild-type littermates. At E16.5, 33% of *Nisch*^*edsn/edsn*^ lungs appeared severely affected (n = 6). This was replicated at E18.5 and P0, whereby 30% (n = 10) and 29% (n = 7) of *Nisch*^*edsn/edsn*^ lungs appeared severely affected respectively. At each time point, the width of airspaces in severely affected *Nisch*^*edsn/edsn*^ mutants was significantly smaller than wild-type tissue (one-way ANOVA: *p* < 0.001), whereas no difference in the number of airspaces was observed. The reduced numbers of *Nisch*^*edsn/edsn*^ progeny are likely a consequence of their lung defect.

Consistent with a number of other adult mouse mutants harbouring defects in embryonic lung generation [22], histological evaluation of adult *edison* lungs revealed enlargement of the airspaces in *Nisch*^*edsn/edsn*^ mice, characteristic of an emphysema-like phenotype (Fig 4E–4H). On average, the airspace width in surviving adult *Nisch*^*edsn/edsn*^ lungs was significantly larger than wild-type tissue (Kruskall-Wallis: *p* < 0.001). Moreover, in *Nisch*^*edsn/edsn*^ lungs quantification of the number of airspaces indicated a significant decrease compared to wild-type (Kruskall-Wallis: *p* < 0.001). Interestingly, the severity of the emphysema-like lung phenotype observed in *Nisch*^*edsn/edsn*^ replicates the severity of the OM phenotype in the ME (Fig 4I–4M). The mean number of airspaces in *Nisch*^*edsn/edsn*^ animals with no OM phenotype was significantly higher compared to *Nisch*^*edsn/edsn*^ animals with bilateral OM (Clear, 200 ± 6.8; Bilateral OM, 104 ± 6.8; Kruskall-Wallis: *p* < 0.001). Similarly, the average airspace width in *Nisch*^*edsn/edsn*^ mice with no OM phenotype was significantly smaller than *Nisch*^*edsn/edsn*^ animals with bilateral OM (Clear, 36.4 ± 1.31 μm; Bilateral OM, 80.4 ± 2.97 μm; Kruskall-Wallis: *p* < 0.001).

Macrophages are the primary phagocytic cells in the lungs. In wild-type lungs, F4/80-positive cells are generally relatively small and rounded (Fig 4N). In contrast, abnormal accumulations of enlarged alveolar macrophages containing coarsely granular material were observed within the lungs of *Nisch*^*edsn/edsn*^ mice (Fig 4O).

### Genetic interaction of *Nisch* and *Itga5*

**Fig 5:**
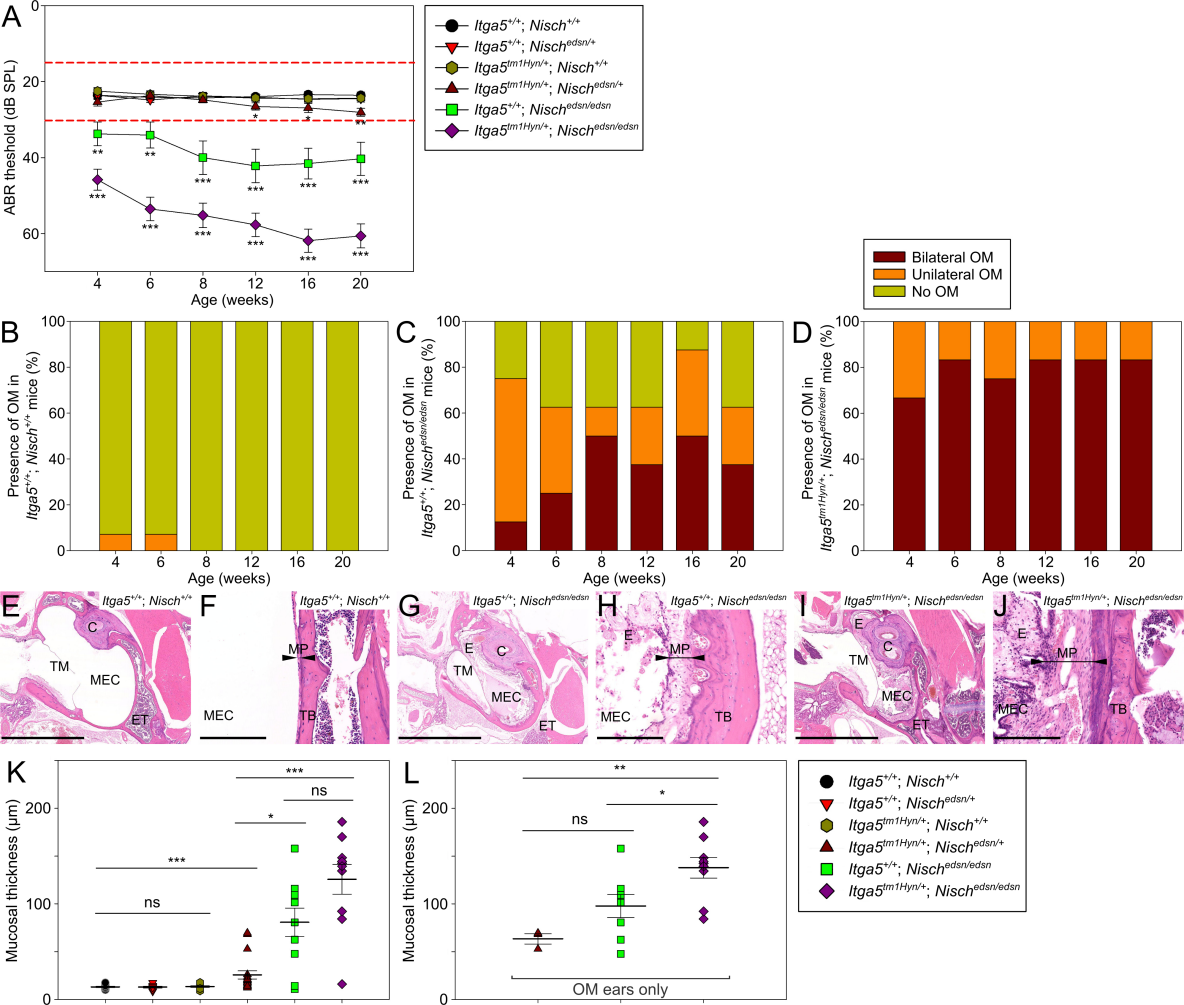
Deficiencies in *Nisch* and *Itga5* exacerbate the otitis media phenotype. **(A)** Click-evoked ABR thresholds across a time course show *Itga5*^*tm1Hyn/+*^; *Nisch*^*edsn/edsn*^ mice exhibit significantly elevated auditory thresholds compared to *Itga5*^*+/+*^; *Nisch*^*edsn/edsn*^ mice. Additionally, a mild late-onset hearing deficit is observed in *Itga5*^*tm1Hyn/+*^; *Nisch*^*edsn/+*^ mice, with onset at 12 wk. Expected ABR threshold range for normal hearing was between 15–30 dB SPL (dashed red lines). *Itga5*^*+/+*^; *Nisch*^*+/+*^ n = 14; *Itga5*^*+/+*^; *Nisch*^*edsn/+*^ n = 14; *Itga5*^*tm1Hyn/+*^; *Nisch*^*+/+*^ n = 15; *Itga5*^*tm1Hyn/+*^; *Nisch*^*edsn/+*^ n = 13; *Itga5*^*+/+*^; *Nisch*^*edsn/edsn*^ n = 8; *Itga5*^*tm1Hyn/+*^; *Nisch*^*edsn/edsn*^ n = 12. **(B-D)** Visual inspection of the tympanic membrane was used as a semi-quantitative measure for the prevalence of OM. (B) *Itga5*^*+/+*^; *Nisch*^*+/+*^ mice show a very low prevalence of unilateral OM at 4 wk and 6 wk only. (C) *Itga5*^*+/+*^; *Nisch*^*edsn/edsn*^ mice show a progressive increase in prevalence of OM, whereas (D) *Itga5*^*tm1Hyn/+*^; *Nisch*^*edsn/edsn*^ mice display a consistently high prevalence of bilateral OM throughout the time course. **(E-J)** H&E stained transverse sections of the MEC and mucoperiosteum, in 20 wk (E, F) *Itga5*^*+/+*^ *Nisch*^*+/+*^, (G, H) *Itga5*^*+/+*^; *Nisch*^*edsn/edsn*^ and (I, J) *Itga5*^*tm1Hyn/+*^; *Nisch*^*edsn/edsn*^ mice. Both *Itga5*^*+/+*^; *Nisch*^*edsn/edsn*^ and *Itga5*^*tm1Hyn/+*^; *Nisch*^*edsn/edsn*^ mice demonstrate chronic inflammation with an exudate. Inflammation of mucosa was more severe in sections from *Itga5*^*tm1Hyn/+*^; *Nisch*^*edsn/edsn*^ ears, with increased polypoid exophytic growths and a thick cellular effusion. **(K)** Blinded assessment of mean mucosal thickness demonstrates significant increases in *Itga5*^*tm1Hyn/+*^; *Nisch*^*edsn/+*^, *Itga5*^*+/+*^; *Nisch*^*edsn/edsn*^ and *Itga5*^*tm1Hyn/+*^; *Nisch*^*edsn/edsn*^ mice compared to wild-type. Both *Itga5*^*+/+*^; *Nisch*^*edsn/edsn*^ and *Itga5*^*tm1Hyn/+*^; *Nisch*^*edsn/edsn*^ mice exhibit significant increases in mucosal thickness compared to *Itga5*^*tm1Hyn/+*^; *Nisch*^*edsn/+*^ mice. *Itga5*^*+/+*^; *Nisch*^*+/+*^ n = 12; *Itga5*^*+/+*^; *Nisch*^*edsn/+*^ n = 8; *Itga5*^*tm1Hyn/+*^; *Nisch*^*+/+*^ n = 10; *Itga5*^*tm1Hyn/+*^; *Nisch*^*edsn/+*^ n = 18; *Itga5*^*+/+*^; *Nisch*^*edsn/edsn*^ n = 10; *Itga5*^*tm1Hyn/+*^; *Nisch*^*edsn/edsn*^ n = 10. **(L)** To account for the disparities in OM prevalence, the mean mucosal thickness was additionally assessed for OM ears only. *Itga5*^*tm1Hyn/+*^; *Nisch*^*edsn/edsn*^ OM ears exhibit significant increases in mucosal thickness compared to *Itga5*^*+/+*^; *Nisch*^*edsn/edsn*^ OM ears. OM only: *Itga5*^*tm1Hyn/+*^; *Nisch*^*edsn/+*^ n = 3; *Itga5*^*+/+*^; *Nisch*^*edsn/edsn*^ n = 8; *Itga5*^*tm1Hyn/+*^; *Nisch*^*edsn/edsn*^ n = 9. C, cochlea; ET, eustachian tube; E, exudate; MEC, middle ear cavity; MP, mucoperiosteum (arrowheads); TB, temporal bone; TM, tympanic membrane. E, G, I scale bar = 2 mm; F, H, J scale bar = 200 μm. ns *P* > 0.05; * *P* < 0.05; ** *P* < 0.01; *** *P* < 0.001. Error bars indicate standard error of mean. A Kruskall-Wallis test was performed followed by Dunn’s multiple comparison tests for post-hoc analysis.

NISCH binds to the cytoplasmic domain of ITGA5 [23], and is thought to regulate its expression [24]. Cross-talk between VEGF and integrins has been shown to be a critical factor in the regulation of angiogenesis and vascularization [25]. Given these findings we examined the genetic interaction between *Nisch* and *Itga5*. *Nisch*^*edsn/+*^ mice and *Itga5*^*tm1Hyn/+*^; *Nisch*^*edsn/+*^ double heterozygotes were intercrossed to produce *Itga5*^*tm1Hyn/+*^; *Nisch*^*edsn/edsn*^ littermates for the study, with *Itga5*^*tm1Hyn/+*^; *Nisch*^*+/+*^ and *Itga5*^*+/+*^; *Nisch*^*edsn/edsn*^ progeny as littermate controls (Fig 5).

*Itga5*^*+/+*^; *Nisch*^*+/+*^, *Itga5*^*+/+*^; *Nisch*^*edsn/+*^ and *Itga5*^*tm1Hyn/+*^; *Nisch*^*+/+*^ mice showed normal click ABR thresholds across the study (Fig 5A). Both *Itga5*^*+/+*^; *Nisch*^*edsn/edsn*^ and *Itga5*^*tm1Hyn/+*^; *Nisch*^*edsn/edsn*^ mice displayed a progressive hearing loss with onset at 4 wk. Interestingly, *Itga5*^*tm1Hyn/+*^; *Nisch*^*edsn/edsn*^ mice exhibited significantly elevated auditory thresholds compared to *Itga5*^*+/+*^; *Nisch*^*edsn/edsn*^ mice, throughout the time course (Kruskall-Wallis: *p* < 0.01). At 20 wk, *Itga5*^*tm1Hyn/+*^; *Nisch*^*edsn/edsn*^ animals were recorded with a mean click ABR threshold of 61 ± 3 dB SPL, compared to 40 ± 4 dB SPL for *Itga5*^*+/+*^; *Nisch*^*edsn/edsn*^ mice. There was a consistently high prevalence of OM in *Itga5*^*tm1Hyn/+*^; *Nisch*^*edsn/edsn*^ mice compared to *Itga5*^*+/+*^; *Nisch*^*edsn/edsn*^ animals (Fig 5C and 5D). *Itga5*^*tm1Hyn/+*^; *Nisch*^*edsn/edsn*^ mice exhibited significantly increased OM prevalence at 4 wk compared to *Itga5*^*+/+*^; *Nisch*^*edsn/edsn*^ mice (Fisher Exact: *p* = 0.026). At 4 wk, 67% of *Itga5*^*tm1Hyn/+*^; *Nisch*^*edsn/edsn*^ mutants had bilateral OM and 33% had unilateral OM (n = 12), whereas in *Itga5*^*+/+*^; *Nisch*^*edsn/edsn*^ mice at 4 wk, 13% had bilateral OM, 63% unilateral OM and 25% showed no OM phenotype (n = 8). By 20 wk, the difference in OM prevalence observed between *Itga5*^*tm1Hyn/+*^; *Nisch*^*edsn/edsn*^ and *Itga5*^*+/+*^; *Nisch*^*edsn/edsn*^ mice was still increased (Fisher Exact: *p* = 0.051). In *Itga5*^*tm1Hyn/+*^; *Nisch*^*edsn/edsn*^ mutants at 20 wk, 83% had bilateral OM and 17% had unilateral OM (n = 12). While, in *Itga5*^*+/+*^; *Nisch*^*edsn/edsn*^ mice at 20 wk, 38% had bilateral OM, 25% unilateral OM and 37% showed no OM phenotype (n = 8). One *Itga5*^*tm1Hyn/+*^; *Nisch*^*+/+*^ mouse (n = 15) displayed unilateral OM at 4 wk, with no other recordings at later time points (S4A Fig).

Histological examination confirmed that *Itga5*^*+/+*^; *Nisch*^*edsn/edsn*^ and *Itga5*^*tm1Hyn/+*^; *Nisch*^*edsn/edsn*^ mice develop chronic OM (Fig 5E–5J). *Itga5*^*tm1Hyn/+*^; *Nisch*^*edsn/edsn*^ mice displayed a more severe mucosal inflammation, with increased polypoid exophytic growths and a thick cellular effusion. Blinded assessment of the mucoperiosteum thickness (Fig 5K) indicated that both *Itga5*^*+/+*^; *Nisch*^*edsn/edsn*^ and *Itga5*^*tm1Hyn/+*^; *Nisch*^*edsn/edsn*^ mice had significant mucosal thickening compared to wild-type littermates (Kruskall-Wallis: *p* = 0.003 and *p* < 0.001 respectively). Additionally, when only OM ears from *Itga5*^*+/+*^; *Nisch*^*edsn/edsn*^ and *Itga5*^*tm1Hyn/+*^; *Nisch*^*edsn/edsn*^ mice were compared (Fig 5L), there was a significant increase in mucosal thickness observed in *Itga5*^*tm1Hyn/+*^; *Nisch*^*edsn/edsn*^ mice (*Itga5*^*+/+*^; *Nisch*^*edsn/edsn*^, 97.8 ± 12.12 μm, n = 8; *Itga5*^*tm1Hyn/+*^; *Nisch*^*edsn/edsn*^, 137.9 ± 10.85 μm, n = 9; Kruskall-Wallis: *p* = 0.026).

Additionally, double heterozygotes (*Itga5*^*tm1Hyn/+*^; *Nisch*^*edsn/+*^) exhibited a mild late-onset hearing loss, with onset at 12 wk (Fig 5A). Visualisation of the tympanic membrane showed this mild hearing loss was associated with a late-onset in the prevalence of OM from 12 wk in *Itga5*^*tm1Hyn/+*^; *Nisch*^*edsn/+*^ mice (S4B Fig). By 20 wk, *Itga5*^*tm1Hyn/+*^; *Nisch*^*edsn/+*^ mice exhibited significantly increased prevalence of OM compared to wild-type littermates (Fisher Exact: *p* = 0.041). In *Itga5*^*tm1Hyn/+*^; *Nisch*^*edsn/+*^ mutants at 20 wk, 31% had unilateral OM and 69% had no OM phenotype (n = 13). Histological examination confirmed that *Itga5*^*tm1Hyn/+*^; *Nisch*^*edsn/+*^ mice develop chronic OM (S4D and S4F Fig). The mucosal inflammation was diffuse and of mild severity, with the presence of a cellular effusion. Blinded assessment of the mucoperiosteum thickness indicated that *Itga5*^*tm1Hyn/+*^; *Nisch*^*edsn/+*^ mice had significant mucosal thickening compared to wild-type littermates (Fig 5K).

### Expression analysis in wild-type, *Nisch*^*edsn/edsn*^ and *Itga5*^*tm1Hyn/+*^; *Nisch*^*edsn/edsn*^ mice

We proceeded to investigate the expression of interacting partners to NISCH, including ITGA5, as well as key downstream effectors in order to relate the underlying mutation to the *edison* phenotype. In addition to binding ITGA5, NISCH has also been shown to interact directly with PAK1 [23,26], and RAC1 [27]. As well as being a downstream effector of PAK1, LIMK1 [28] also interacts directly with NISCH. In addition PAK controls NF-κB activation [29] and RAC1 leads to activation of NF-κB [30,31]. We thus performed IHC expression analysis of NISCH, ITGA5, phosphorylated-PAK1 (p-PAK1), phosphorylated-LIMK1/2 (p-LIMK1/2), RAC1 and NF-κB p65 on ME epithelia from wild-type, *Nisch*^*edsn/edsn*^ and *Itga5*^*tm1Hyn/+*^; *Nisch*^*edsn/edsn*^ mice. In addition, we assessed protein expression in ME epithelia by western blot analysis for each of these proteins.

**Fig 6:**
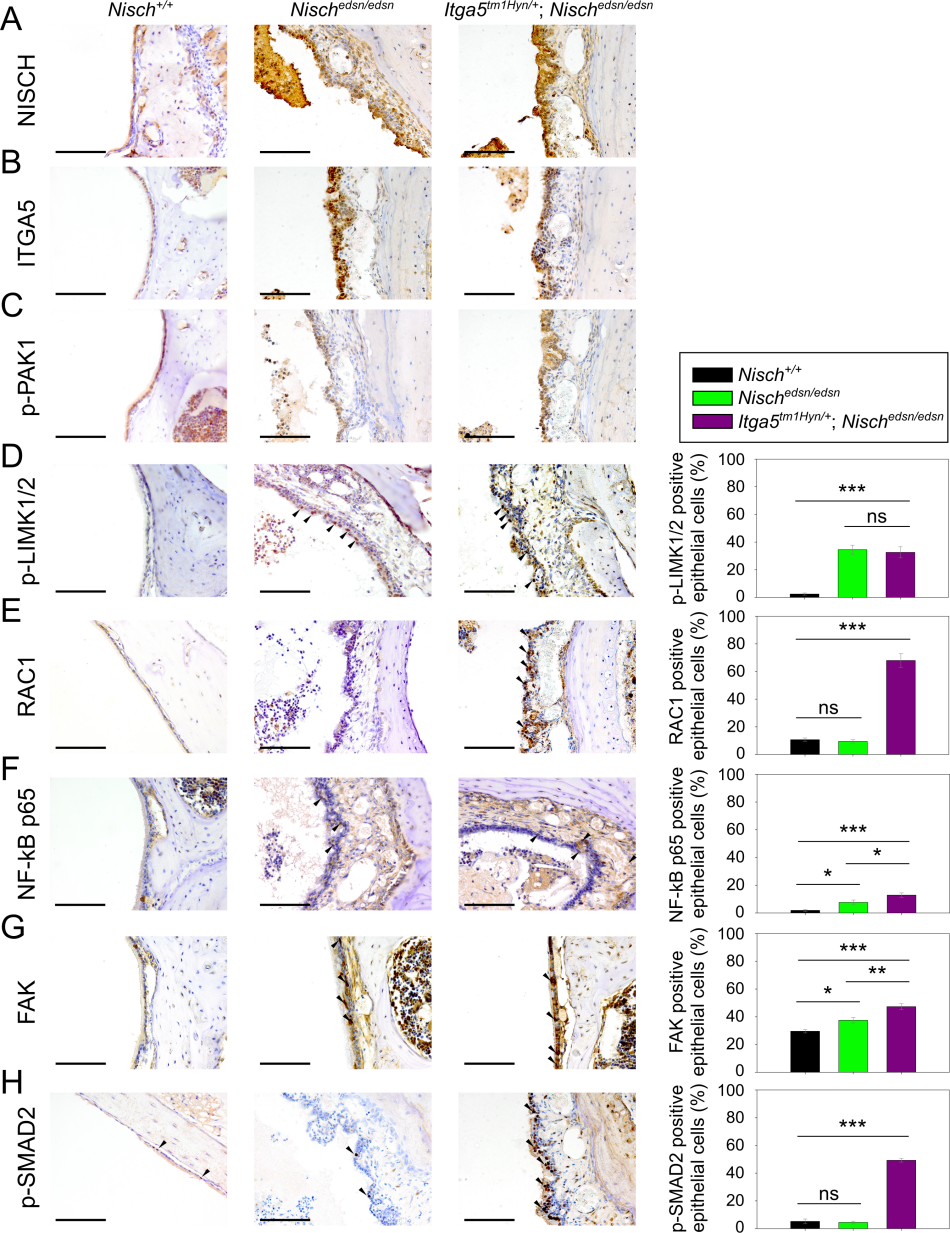
Protein expression analysis of NISCH interacting partners and downstream pathways in middle ear by immunohistochemistry. Middle ear sections of *Nisch*^*+/+*^, *Nisch*^*edsn/edsn*^ and *Itga5*^*tm1Hyn/+*^; *Nisch*^*edsn/edsn*^ mice at 3 wk, stained with **(A)** NISCH, **(B)** ITGA5, **(C)** p-PAK1, **(D)** p-LIMK1/2, **(E)** RAC1, **(F)** NF-κB p65, **(G)** FAK and **(H)** p-SMAD2 antibodies. To quantify the results middle ear epithelial cells in wild-types and mutants were counted in four middle ears from each genotype. Scale bar = 100 μm. ns *P* > 0.05; * *P* < 0.05; ** *P* < 0.01; *** *P* < 0.001. Error bars indicate standard error of mean. The data was analysed by one-way ANOVAs and Holm-Sidak’s multiple comparison procedures for post-hoc testing.

**Fig 7:**
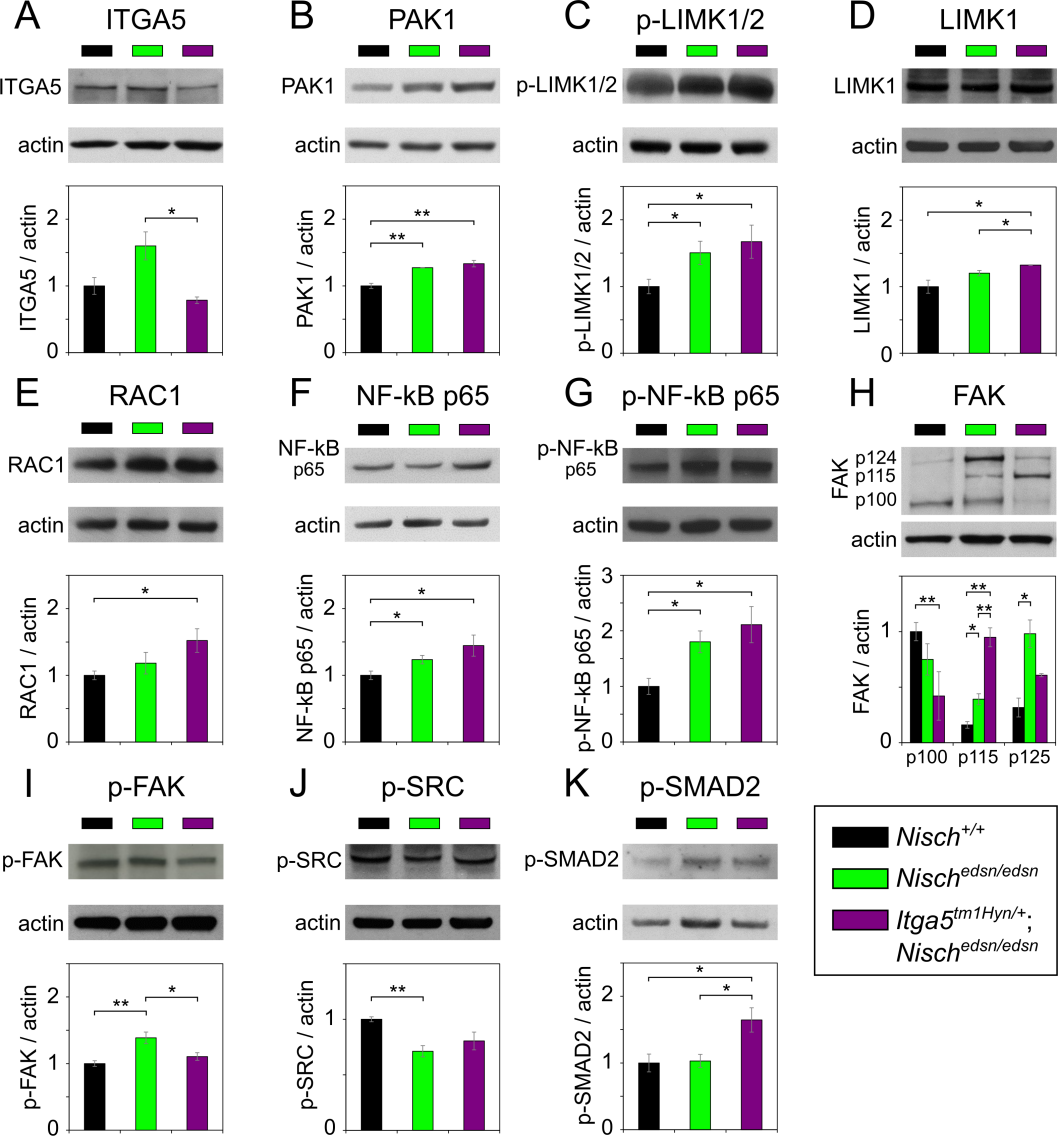
Protein expression analysis of NISCH interacting partners and downstream pathways in middle ear by western blot. Middle ear epithelial cells extracts of *Nisch*^*+/+*^, *Nisch*^*edsn/edsn*^ and *Itga5*^*tm1Hyn/+*^*; Nisch*^*edsn/edsn*^ mice at 8 wk, probed with **(A)** ITGA5, **(B)** PAK1, **(C)** p-LIMK1/2, **(D)** LIMK1, **(E)** RAC1, **(F)** NF-κB p65, **(G)** p-NFκB p65, **(H)** FAK, **(I)** p-FAK, **(J)** p-SRC and **(K)** p-SMAD2 antibodies. The results presented are from four independent experiments for all the antibodies except for ITGA5, PAK1, LIMK1, p-NFκB p65 and FAK, where three independent experiments were used to present the data. ns *P* > 0.05; * *P* < 0.05; ** *P* < 0.01; *** *P* < 0.001. Error bars indicate standard error of mean. A Student’s t-test was performed to analyse the data.

IHC labelling for NISCH, ITGA5 and p-PAK1 was observed in ME epithelial cells with similar patterns of expression in *Nisch*^*+/+*^, *Nisch*^*edsn/edsn*^ and *Itga5*^*tm1Hyn/+*^; *Nisch*^*edsn/edsn*^ mice. No obvious differences in localisation were observed for these three proteins, although staining was stronger in *Nisch*^*edsn/edsn*^ and *Itga5*^*tm1Hyn/+*^; *Nisch*^*edsn/edsn*^ mice (Fig 6A–6C). Western analysis of ME epithelial cell lysates showed raised levels of ITGA5 in *Nisch*^*edsn/edsn*^ mice but not significantly different compared to wild-type (t-test: *p* = 0.071) and not surprisingly a significant decline in levels between *Nisch*^*edsn/edsn*^ and *Itga5*^*tm1Hyn/+*^; *Nisch*^*edsn/edsn*^ mice (t-test: *p* = 0.020) (Fig 7A). PAK1 protein levels were significantly raised in both *Nisch*^*edsn/edsn*^ (t-test: *p* = 0.002), and *Itga5*^*tm1Hyn/+*^; *Nisch*^*edsn/edsn*^ mice (t-test: *p* = 0.006) when compared to wild-type, but not between *Nisch*^*edsn/edsn*^ and *Itga5*^*tm1Hyn/+*^; *Nisch*^*edsn/edsn*^ mice (t-test: *p* = 0.282) (Fig 7B).

Available antibodies for p-LIMK1 were ineffective. However, we carried out expression analyses using a p-LIMK1/2 and LIMK1 antibody. IHC analysis of p-LIMK1/2 expression in ME epithelial cells revealed nuclear localisation and increased expression from both *Nisch*^*edsn/edsn*^ and *Itga5*^*tm1Hyn/+*^; *Nisch*^*edsn/edsn*^ mice (one-way ANOVA: *p* < 0.001) compared to wild-type. However, no significant difference was observed between ME epithelial cells in *Nisch*^*edsn/edsn*^ and *Itga5*^*tm1Hyn/+*^; *Nisch*^*edsn/edsn*^ mice (one-way ANOVA: *p* = 0.704) (Fig 6D). Similarly, protein levels of p-LIMK1/2 in ME epithelial cells were significantly raised in *Nisch*^*edsn/edsn*^ (t-test: *p* = 0.039) and *Itga5*^*tm1Hyn/+*^; *Nisch*^*edsn/edsn*^ (t-test: *p* = 0.038) compared to wild-type. No difference was detected between *Nisch*^*edsn/edsn*^ and *Itga5*^*tm1Hyn/+*^; *Nisch*^*edsn/edsn*^ samples (t-test: *p* = 0.598), consistent with the observations from immunohistochemistry (Fig 7C). In addition, levels of LIMK1 in ME epithelial cells were also significantly raised in *Itga5*^*tm1Hyn/+*^; *Nisch*^*edsn/edsn*^ compared to wild-type (t-test: *p* = 0.030) and *Nisch*^*edsn/edsn*^ (t-test: *p* = 0.041) mice (Fig 7D).

IHC analysis of RAC1 demonstrated nuclear localisation and increased expression from *Itga5*^*tm1Hyn/+*^; *Nisch*^*edsn/edsn*^ mice compared to wild-type (one-way ANOVA: *p* < 0.001) (Fig 6E). Moreover, in agreement with these observations, protein levels of RAC1 were significantly raised in *Itga5*^*tm1Hyn/+*^; *Nisch*^*edsn/edsn*^ mice (t-test: *p* = 0.033), compared to wild-type (Fig 7E).

IHC analysis of NF-κB p65 expression showed nuclear localisation and raised levels of protein in both *Nisch*^*edsn/edsn*^ and *Itga5*^*tm1Hyn/+*^; *Nisch*^*edsn/edsn*^ mice compared to wild-type (one-way ANOVA: *p* = 0.017 and *p* <0.001 respectively). Moreover, there was a significant enhancement of NF-κB labelling in the double mutant compared to *Nisch*^*edsn/edsn*^ (one-way ANOVA: *p* = 0.026) (Fig 6F). In agreement with IHC, we found significantly higher levels of NF-κB p65 by western blot in both mutants compared to wild-type (t-test: *p* = 0.039 and *p* = 0.041) (Fig 7F). We also examined levels of activated NF-κB p65 by western blot employing an antibody that recognises phosphorylated-NF-κB [Ser 276] p65 (p-NF-κB p65) and detected significantly higher levels of the activated protein in both mutants compared to wild-type (t-test: *p* = 0.030 and *p* = 0.035) (Fig 7G).

In addition, we investigated the expression of two pathways that are implicated in the development of chronic OM (see Introduction), or are regulated by NISCH interacting partners. First, we evaluated levels of focal adhesion kinase (FAK) and SRC. Cross-talk between integrins and VEGF is a critical factor in the regulation of angiogenesis, vascularisation and vascular permeability [25]. Integrins regulate VE-cadherin via the activation of SRC, and FAK catalytic activity is required when α5β1 integrin stimulates SRC activation through FAK phosphorylation. FAK inhibition prevents VEGF-stimulated vascular permeability underlining the importance of FAK activity in the regulation of adherens junctions [32]. Tyr 397 in human FAK becomes phosphorylated upon integrin engagement and creates a binding site for SRC. This results in release of the inactive conformation of SRC (Tyr 527) and leads to autophosphorylation of SRC on Tyr 416. Activated SRC further phosphorylates FAK on additional residues, one of which is Tyr 576. The activated FAK-SRC complex then initiates multiple downstream signalling pathways [33,34]. IHC of ME epithelial cells showed there was increased expression of total FAK in both *Nisch*^*edsn/edsn*^ and *Itga5*^*tm1Hyn/+*^; *Nisch*^*edsn/edsn*^ compared to wild-type mice (one-way ANOVA: *p* = 0.015 and *p* < 0.001 respectively). A significant difference was also observed between *Nisch*^*edsn/edsn*^ and *Itga5*^*tm1Hyn/+*^; *Nisch*^*edsn/edsn*^ ears (one-way ANOVA: *p* = 0.005) (Fig 6G).

To study protein levels of FAK we used an antibody raised against the last 50 amino acids at the C-terminal of the human protein. There are nine known mouse isoforms of FAK (http://www.uniprot.org/uniprot/P34152) produced by alternative promoter usage and alternative splicing and the antibody is potentially able to detect six of them. We detected three main bands in ME epithelial cell lysates at 124, 115 and 100kDa. The full-length canonical isoform is 124kDa. We detected significantly increased levels of the full length 124kDa FAK1 protein in *Nisch*^*edsn/edsn*^ mutants compared to wild-type (t-test: *p* = 0.014). The level of the 115kDa form was significantly raised in *Nisch*^*edsn/edsn*^ (t-test: *p* = 0.014) and *Itga5*^*tm1Hyn/+*^; *Nisch*^*edsn/edsn*^ (t-test: *p* = 0.001) mice compared to wild-type, while the 100kDa form was the main isoform detected in wild-type samples. A significant difference was detected in the levels of the 100kDa between wild-type and *Itga5*^*tm1Hyn/+*^; *Nisch*^*edsn/edsn*^ (t-test: *p* = 0.0005) (Fig 7H). Furthermore we used phosphorylated-FAK (p-FAK) [Y576] and phosphorylated-SRC (p-SRC) [Y527] antibodies to test the activity of the FAK-SRC complex in middle ear epithelia. Using a p-FAK [Y576] antibody, which recognises activated FAK, we detected an increase in the activated FAK in the *Nisch*^*edsn/edsn*^ tissue compared to the wild-type ME epithelial cell lysate (t-test: *p* = 0.007) (Fig 7I). Not surprisingly, in the double mutant *Itga5*^*tm1Hyn/+*^; *Nisch*^*edsn/edsn*^, levels of activated FAK were not significantly different to wild-type. Using a p-SRC [Y527] antibody, which recognises inactive SRC, we detected complementary results to that seen with activated FAK. We found reduced levels of protein in *Nisch*^*edsn/edsn*^ ME lysates compared to the wild types (t-test: *p* = 0.002) but no differences between wild-type and *Itga5*^*tm1Hyn/+*^; *Nisch*^*edsn/edsn*^ (Fig 7J).

Finally, we proceeded to evaluate activation of the TGF-β pathway during chronic ME disease by assessment of phosphorylated-SMAD2 (p-SMAD2). IHC analysis of p-SMAD2 revealed significantly raised levels in *Itga5*^*tm1Hyn/+*^; *Nisch*^*edsn/edsn*^ mice compared to wild-type (one-way ANOVA: *p* < 0.001). However, there was no significant difference between *Nisch*^*edsn/edsn*^ and wild-type mice (one-way ANOVA: *p* = 0.680) (Fig 6H). In addition, protein levels of p-SMAD2 in ME epithelial cells were significantly higher in *Itga5*^*tm1Hyn/+*^; *Nisch*^*edsn/edsn*^ mice compared to wild-type (t-test: *p* = 0.029) or *Nisch*^*edsn/edsn*^ (t-test: *p* = 0.024) mice. Again no difference was detected between wild-type and *Nisch*^*edsn/edsn*^ mice (t-test: *p* = 0.864) (Fig 7K).

IHC analysis of this suite of proteins in airway epithelia revealed many similarities to that seen in ME epithelia (S5 Fig). Levels of NISCH appeared to be raised in both *Nisch*^*edsn/edsn*^ and *Itga5*^*tm1Hyn/+*^; *Nisch*^*edsn/edsn*^ mice compared to wild-type. Furthermore, increased epithelia expression of p-LIMK1/2 in both *Nisch*^*edsn/edsn*^ and *Itga5*^*tm1Hyn/+*^; *Nisch*^*edsn/edsn*^ mice mirrored the findings in ME epithelia. Similarly, RAC1 epithelial levels were significantly raised in *Itga5*^*tm1Hyn/+*^; *Nisch*^*edsn/edsn*^ mice, while NF-κB and FAK expression was raised in both *Nisch*^*edsn/edsn*^ and *Itga5*^*tm1Hyn/+*^; *Nisch*^*edsn/edsn*^ mice. However, we observed no significant changes in p-SMAD2 levels in mutant airways. In contrast, in lung tissue we observed very few significant changes in protein levels by western blot analysis (S5 Fig). These results may reflect the complexity of tissues and cell types isolated from dissected material and the visible mesenchymal expression for many of these proteins in airway tissue.

## Discussion

In a large-scale ENU mutagenesis screen we recovered a new recessive mouse model of chronic OM, *edison*. The *edison* mutant carries a Leu972Pro change in the Nischarin gene. *Nisch*^*edsn/edsn*^ homozygotes display a progressive middle ear disease with 56% of mice displaying bilateral OM by 20 weeks and elevated ABR thresholds of 20–30 dB SPL indicative of a conductive hearing loss. We derived an additional ENU allele (*Nisch*^*V33A*^) in the Nischarin gene which also presents with progressive chronic OM, but where ABR thresholds were only very moderately increased and at 12 weeks only 10% of the mice had bilateral OM. It appears that the *Nisch*^*V33A*^ allele is severely hypomorphic. Compound heterozygotes of the *edison* and *Nisch*^*V33A*^ alleles showed an intermediate non-complementing OM phenotype. We did not identify any sensorineural element to the hearing loss in the *Nisch*^*edsn/edsn*^ mutant.

The chronic OM in *Nisch*^*edsn/edsn*^ mice is exemplified by exudate within the ME cavity and a thickened mucoperiosteum and polypoid exophytic growths, sometimes associated with an inflamed tympanic membrane. Serous or granulocyte-rich effusions were observed, but as the mice aged a thick effusion was predominately observed rich in macrophages and PMNs. Examination of ME exudates for upregulation of both inflammatory genes and hypoxia genes found that both *Il-1b* and *Tnfa* were both upregulated, as were *Hif1a* and the HIF responsive gene *Vegfa*. This is similar to the findings reported for other mouse models of chronic OM, such as the *Jeff* [8], *Junbo* [9] and *Tgif1* [35] mutants. Both the middle ear and the lungs have substantial similarities in structure and function [36] and, intriguingly, we identified a lung defect in *Nisch*^*edsn/edsn*^ mice. During embryonic development, we found a significant reduction in airspace width, while in the adults, we observed an emphysema-like phenotype with enlarged airspace width and a reduced number of airspaces. *In utero*, the network of airways is generated first followed by formation of the gas-exchanging units (alveoli) that develop from the distal ends of the small airways. As a consequence disruption to lung development often results in narrower airspaces in embryonic lungs but enlarged airspaces post-natally because of insufficient generation of alveoli. These lung abnormalities likely account for the deficit of *Nisch*^*edsn/edsn*^ mice recovered from the various crosses that we report.

The discovery of the involvement of Nischarin in the development of inflammatory middle ear disease identifies a novel gene and associated pathways that are involved in OM. This led us to explore the intersection with known pathways of OM [11,13,14] and the downstream signalling mechanisms that lead to the OM phenotype. Nischarin is a highly conserved protein across mammalian species, consisting of an N-terminal phox homology (PX) domain, 6 putative leucine-rich repeats, a coiled-coil domain, an alanine/proline-rich region and a long C-terminal region. NISCH has a multitude of interacting partners, including ITGA5 [37], PAK1 [26], Rac1 [27,38], LIMK1 [28], Rab14, PI3P [38], and LKB1 [39]. Association of NISCH with these interacting partners underlines its broad impact on the regulation of cell motility, cell invasion, vesicle maturation, as well as its role as a tumour suppressor [28,37,38,40]. Most notably, the binding of NISCH to ITGA5 [37] is thought to mediate the translocation of ITGA5 from the cell membrane to endosomes [24] thus regulating ITGA5 levels. As we discuss above, integrins have been shown to play a critical role in modulating VEGF-induced angiogenesis and vascularization [25]. These pathways thus intersect with the hypoxia-response pathways mediated by HIF-1a which have been demonstrated to be involved with the development of chronic OM in the *Junbo* and *Jeff* models [14]. Hypoxia leads to upregulation of VEGFA and downstream pathway genes resulting in VEGF-induced angiogenesis and vascular leak. VEGFR inhibitors moderated angiogenesis and lymphangiogenesis in the *Junbo* mouse. For these reasons we sought to explore the role of ITGA5 in the *edison* mutant, and also to characterise the responses of downstream pathways which may illuminate the mechanisms of OM development in *edison*.

We found a strong genetic interaction between *edison* and *Itga5* mutants. *Itga5*^*tm1Hyn/+*^; *Nisch*^*edsn/edsn*^ double mutants compared to *Nisch*^*edsn/edsn*^ mice showed significantly elevated ABR thresholds as well as a very significant raised frequency of bilateral OM in mice from 4 weeks onwards. In summary, the OM was more highly penetrant from an earlier age, and commensurately less progressive. Given the interactions of ITGA5 and NISCH, along with the interactions of NISCH with diverse molecules involved in LIMK1 and NF-κB signalling, we sought to interpret this genetic interaction in the context of known signalling pathways and interactions downstream of NISCH. Moreover, in developing a mechanistic model, we took into account the reports that interaction of ITGA5 with NISCH affects some of the downstream interactions of the NISCH molecule itself.

RAC1 signalling regulates disparate cellular functions mediated through a variety of effector proteins [30,31]. PAK1 is a key downstream effector of RAC1 [41,42], with binding of RAC1 leading to activation of PAK1 [43]. NISCH represses this pathway, and NISCH has been shown to block RAC1 induced cell migration through binding to PAK1; interaction with NISCH strongly inhibits the kinase activity of PAK1 [23]. RAC1 activation of PAK1 enhances the interaction between NISCH and PAK1, while notably expression of ITGA5 also increases the association between NISCH and PAK1 [23]. LIMK1 is a downstream effector of PAK1 [44] and has been shown to be involved in vascular permeability [45]. NISCH is also an interacting partner with LIMK1, regulating cell invasion through repression of the LIMK1-cofilin pathway [28]. NISCH also regulates PAK1-independent RAC1 signalling through direct interaction with RAC1 [27]. RAC1 stimulates the phosphorylation and degradation of IkB and up-regulates NF-κB [46,47]. Overexpression of NISCH has been shown to suppress the ability of RAC1 to stimulate NF-κB activation [27]. The *Junbo* OM mutant carries a mutation in the *Evi1* gene, and it is noteworthy that EVI1 is a negative-feedback regulator of NF-κB [13]. The mutation in *Junbo* leads to activation of NF-κB and inappropriate regulation of the inflammatory response [13].

We have assessed the expression of the critical genes within these pathways in the ME epithelia of wild-type, *Nisch*^*edsn/edsn*^ and *Itga5*^*tm1Hyn/+*^; *Nisch*^*edsn/edsn*^ mice. First, we observed that levels of ITGA5 were raised in *Nisch*^*edsn/edsn*^ mice though not significantly, reflecting the role of NISCH in regulating ITGA5 levels. Not surprisingly, ITGA5 was significantly reduced compared to *edison* mice in the double mutant, *Itga5*^*tm1Hyn/+*^; *Nisch*^*edsn/edsn*^. We found significantly raised levels of activated p-PAK1 in both mutant lines compared to wild-type, which was mirrored by downstream raised levels of p-LIMK1/2 in both *Nisch*^*edsn/edsn*^ and *Itga5*^*tm1Hyn/+*^; *Nisch*^*edsn/edsn*^ mice. Raised levels of p-LIMK1/2 were also reflected in the IHC assessment. LIMK1 levels were also raised in *Nisch*^*edsn/edsn*^ *Itga5*^*tm1Hyn/+*^ mice. We were unable to assess directly levels of p-LIMK1 due to the ineffectiveness of available antibodies. While RAC1 protein levels were not affected in *Nisch*^*edsn/edsn*^ mice, they were significantly raised in the double mutant, *Itga5*^*tm1Hyn/+*^; *Nisch*^*edsn/edsn*^ mice which was mirrored in the IHC assessment. We also found by both IHC and western analysis that NF-κB levels were raised in both *Nisch*^*edsn/edsn*^ and *Itga5*^*tm1Hyn/+*^; *Nisch*^*edsn/edsn*^ mice compared to wild-type. Moreover, there is evidence from IHC that the effect of the two mutations are additive and that the double mutant shows a significantly higher level of NF-κB expression compared to *Nisch*^*edsn/edsn*^ mice. While the protein analysis shows a similar trend the differences between *Nisch*^*edsn/edsn*^ and *Itga5*^*tm1Hyn/+*^; *Nisch*^*edsn/edsn*^ mice are not significant.

**Fig 8:**
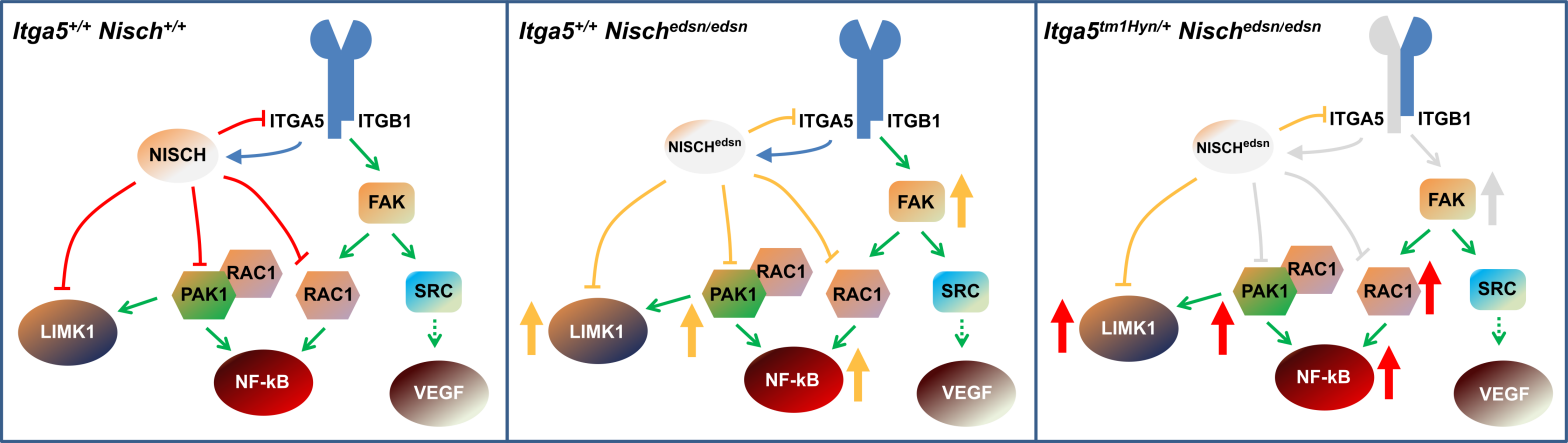
Proposed mechanism. Model of the mechanism of action in wild-type mice (*Itga5*^*+/+*^*; Nisch+*^*/+*^**),** during the onset OM in *edison* mice (*Itga5*^*+/+*^*; Nisch*^*edsn/edsn*^**)** and in double mutants (*Itga5*^*tm1Hyn/+*^*; Nisch*^*edsn/edsn*^). Nisch, Nischarin; Nisch^edsn^, Nischarin with the *edison* mutation; ITGA5, integrin α5; ITGB1, integrin β1; FAK, Focal adhesion kinase; PAK1, p21-activated kinase 1; LIMK1, LIM domain kinase 1; Rac1, Ras-related C3 botulinum toxin substrate 1; NF-κB, nuclear factor kappa-light-chain-enhancer of activated B cells; SRC, Proto-oncogene tyrosine-protein kinase; VEGF, Vascular endothelial growth factor. Red capped lines represent inhibition, yellow capped lines represent reduced inhibition and grey capped lines represent low inhibition. The blue arrow indicates the role of ITGA5 in enhancing binding of NISCH to PAK1. Green arrows indicate direct activation of downstream members of the pathway and dashed green arrows indicate indirect activation. Grey arrows indicate slightly raised levels of proteins, while yellow indicates raised levels and red indicates highly raised levels.

Overall, our analysis indicates that the *edison* mutation leads to activation of PAK1 and PAK1-independent RAC1 pathways with increased levels of NF-κB and p-LIMK1/2 each of which may lead to inflammatory and vascular permeability effects. In addition, a combination of mutations in NISCH and ITGA5 can lead to an exacerbation of raised protein levels which may underlie the more severe phenotype seen in the double mutant, *Itga5*^*tm1Hyn/+*^; *Nisch*^*edsn/edsn*^. This may reflect the role of ITGA5 in enhancing binding of NISCH to PAK1, and provides us with a model of the mechanism underlying the *Nisch*^*edsn/edsn*^ and *Itga5*^*tm1Hyn/+*^; *Nisch*^*edsn/edsn*^ phenotypes (Fig 8). We surmise that impairing function of NISCH leads to derepression of RAC1 pathways, while reducing levels of ITGA5 in combination with impaired NISCH leads to further derepression and activation of downstream pathways manifested in the more severe phenotype. However, we did not observe significantly raised levels of RAC1 in the single mutant. Nevertheless, we found raised levels of NF-κB that may reflect activation of PAK1 independent pathways.

ITGA5 is known to be involved with the activation of SRC protein tyrosine kinase, as well as FAK, both of which are involved with mediating VEGF induced vascular leak [32]. ITGA5 can mediate its effects by phosphorylation of FAK and binding of activated FAK to SRC leading to conformational SRC activation [33]. Alternatively, for example in the context of neuroblastoma cell motility, FAK is required for integrin α5β1-mediated SRC phosphorylation [34]. We thus investigated FAK and SRC levels in the mutant mice, focusing on activated FAK and inactive SRC. We found total levels of the full length isoform of the FAK protein raised in *Nisch*^*edsn/edsn*^ mice. However, in *Itga5*^*tm1Hyn/+*^; *Nisch*^*edsn/edsn*^ mice, full length FAK protein was not significantly different to wild-type. Importantly, we detected raised levels of activated FAK along with reduced levels of inactive SRC in *Nisch*^*edsn/edsn*^ mice. These changes may contribute to angiogenesis and vascular permeability through upregulation of VEGF signalling pathways.

The genes mutated in previously characterised OM models (*Junbo*, *Jeff* and *Tgif1*), have been reported to regulate the TGF-β signalling pathway [11,12,35]. There is considerable cross-talk between TGF-β signalling and hypoxia pathways and mutations that perturb the TGF-β pathway might be expected to perturb hypoxia responses with downstream consequences on VEGFA and VEGF signalling, as is observed in the mutants studied [14]. We discuss above the raised levels of HIF-1a and VEGFA found in the *edison* mutant. We thus assessed TGF-β signalling (in *edison*), and found that p-SMAD2 levels are significantly raised in *Itga5*^*tm1Hyn/+*^; *Nisch*^*edsn/edsn*^ mice, but not in *Nisch*^*edsn/edsn*^. The lack of raised p-SMAD2 levels in the *Nisch*^*edsn/edsn*^ mouse suggests that activation of TGF-β signalling is not a primary event underlying the development of chronic OM in the *edison* mouse. Rather, the upregulation of p-SMAD2 in the double mutant may reflect the very severe inflammatory state of the middle ear leading to activation of TGF-β pathways.

In summary (see Fig 8), we conclude that mutations in Nischarin impact upon PAK-dependent and PAK-independent RAC pathways with downstream signalling effects on LIMK1 and NF-κB. Moreover, the interplay between Nischarin and ITGA5 likely underlies impacts on FAK and SRC signalling which may lead to VEGF mediated vascular leak. The combined effects on LIMK1, NF-κB and FAK signalling can account for the observed inflammatory changes in the *edison* middle ear, which along with vascular leak and middle ear exudate, produce a chronic OM. Together, the pathways changes we describe provide a mechanism underlying the observed phenotypic changes in *edison*. Moreover, these studies further enhance our knowledge of the relevant genetic pathways that contribute to middle ear inflammatory disease, as well as a panel of new genes that are candidates for genetic susceptibility to chronic OM in the human population.

## Materials and methods

### Ethics statement

Mice were bred and maintained by Mary Lyon Centre, MRC Harwell and were housed in specific-pathogen free conditions. All animal experimentation was approved by the Animal Welfare and Ethical Review Body at MRC Harwell (License Numbers: 30/3015 and 30/3280). The humane care and use of mice in this study was under the authority of the appropriate UK Home Office Project License.

### Phenotype-driven ENU-mutagenesis screen

The founder mouse carrying the *edison* mutation was generated in a large-scale phenotype-driven ENU mutagenesis program at MRC Harwell [20]. Briefly, Male C57BL/6J mice were mutagenized and mated to C3H.*Pde6b+* females (a C3H stock that does not carry the retinal degeneration allele *Pde6b*^*rd*^). G_3_ offspring were screened for a variety of abnormalities, including deafness and vestibular dysfunction. The *edison* founder was identified due to lack of a Preyer reflex when presented with a calibrated 20 kHz, 90 dB SPL tone burst via a click-box test.

### Mapping

The founder *edison* mouse was maintained by repeated outcrossing to C3H/HeH and intercrossing to produce homozygous mutant progeny, identified by the lack of a Preyer reflex. For linkage analysis genomic DNA from 13 affected mice were screened with 63 strain specific SNP markers spaced equidistantly across the genome using the Pyrosequencing SNP genotyping system (QIAGEN). Additional SNP markers were used within linked regions to further fine map the causal mutation. Markers and primer sequences are available on request from the authors.

### Whole-genome sequencing

Genomic DNA from a single affected *edison* mouse was sent for next-generation sequencing (High-Throughput Genomics, WTCHG). Identification, analysis and dissemination of sequence variant information identified through whole-genome sequencing were achieved with the tools provided by the MRC Harwell Biocomputing custom sequence analysis pipeline. Sequence reads were mapped to the NCBI37/mm9 assembly of the reference mouse genome. Within the critical interval, the mean read depth was 8.17 and the read coverage was 99.64%. Sequence variants were identified and were subsequently categorised into those which occurred within exons and splice donor/acceptor sites, and were not known or strain-specific variants. This led to the identification of a T to C base substitution within exon 14 of *Nisch*, which is predicted to cause a Leu972Pro missense change in NISCH protein. This sequence variant was validated using Sanger sequencing (Source BioScience, UK). Affected and unaffected mice were genotyped using the LightScanner SNP genotyping system (Idaho Technology Inc., USA) to confirm the association of genotype to phenotype. In all cases the genotype correlated with the phenotype.

### Gene-driven identification of an additional *Nisch* allele

DNA from the MRC Harwell ENU-DNA sperm archive (http://www.har.mrc.ac.uk/services/archiving-distribution/enu-dna-archive) was screened with the LightScanner platform (Idaho Technology Inc., USA). Briefly, male C57BL/6J mice were treated with ENU and crossed to C3H/HeH females. F_1_ progeny (C3H/HeH.C57BL/6J) were rederived and male F_1_ animals had sperm and DNA samples taken for archiving. Ten exons of *Nisch* were screened in DNA from ~10,000 F_1_ ENU mutagenised animals and potential mutations confirmed with Sanger sequencing (Source BioScience, UK).

### Genetic background

All *edison* mice used for phenotyping were congenic on a C3H/HeH background. They were backcrossed for at least ten generations. The *Nisch*^*V33A*^ strain was rederived by *in vitro* fertilisation of C57BL/6J oocytes with F_1_ sperm from the MRC Harwell ENU-DNA sperm archive and maintained on a mixed C3H/HeH and C57BL/6J genetic background. *Nisch*^*V33A/+*^ mice were intercrossed for phenotypic analysis and crossed to congenic *Nisch*^*edsn/+*^ mice for complementation testing. Cryopreserved *Itga5*^*tm1Hyn*^ mutant sperm was imported from the Jackson Laboratory (Stock No. 002274) [48] and the colony was rederived using *in vitro* fertilisation of C57BL/6J oocytes. *Itga5*^*tm1Hyn*^ mice had been backcrossed 4–5 generations onto a C3H/HeH background, before crossing to congenic *Nisch*^*edsn*^ mice for phenotypic analysis.

### Genotyping

Genotyping for *edison* mice was performed using an allelic discrimination assay with primers 5’-GGC AGC ACA AAG ATG GCG GTA AC-3’ and 5’-AAC TGC CGC AAC CGC AAC A-3’ and labelled probes 5’-[6-FAM]_AGC AGC TCG AGC ACA T-3’ (*edsn*) and 5’-[TET]_CAG CTC GGG CAC ATG-3’ (Wild-type). The Applied Biosystems 7900HT Fast System (Applied Biosystems, USA) was used for amplification and analysis. To genotype *Nisch*^*V33A*^ mice, PCR amplification was performed with primers 5’-GAC TGA GTA CCT TGC AGC TA-3’ and 5’-CTG TAA CGG TGT TTG ATC GTC-3’ and an unlabelled probe 5’-CCC TTT AGG CTT ATG TCA TCC AGG TTA C_[SpC3]-3’. The LightScanner System (Idaho Technology Inc., USA) was used for subsequent unlabelled probe genotyping analysis. *Itga5*^*tm1Hyn*^ mice were genotyped with mutant and control specific primers, as described on the Jackson Laboratory Mice Database (http://jaxmice.jax.org/strain/002274).

### Skull morphology and radiography

Analysis of skulls from 20 wk mice was performed using a Faxitron Mx-20 DC-4 specimen X-ray System. ImageJ software was used to measure the skull length, nasal bone length, frontal bone length, parietal bone length and skull width. Allometric comparisons were performed against skull length with at least 12 mice of each genotype.

### Auditory brainstem response

Mice were anesthetized and hearing thresholds determined using ABR, as previously described [7]. The ABR threshold was measured for each ear. Click-evoked hearing assessments for *edison* mice were conducted at 3, 4, 6, 8, 12, 16 and 20 wk with cohorts containing at least 14 mice of each genotype at each time point. Frequency-specific (8, 16, and 32 kHz) analysis of auditory function for *edison* mice was conducted over a longitudinal time course at 4, 6, 8, 12 and 20 wk with 5 mice of each genotype. At least 10 *Nisch*^*V33A*^ mice were used for click-evoked ABR analysis across a longitudinal time course at 4, 6, 8 and 12 wk. Finally, click-evoked analysis of *Itga5*^*tm1Hyn*^ *Nisch*^*edsn*^ hearing thresholds were measured across a longitudinal time course at 4, 6, 8, 12, 16 and 20 wk with at least 8 mice of each genotype.

### Histology

Mouse 3, 4, 6, 8, 12, 16 and 20 wk heads from *Nisch*^*+/+*^, *Nisch*^*edsn/+*^ and *Nisch*^*edsn/edsn*^ mice were fixed for 48 hours in 10% neutral buffered formaldehyde, decalcified in D.F.B decalcifying agent (Kristensen; Pioneer Research Chemicals) for 72 hours and embedded in paraffin following routine procedures. Perinatal heads were processed in the same manner without the decalcification steps. Four-micrometre-thick sections were obtained, de-paraffinized in xylene substitute and rehydrated via a graded ethanol. For morphological observations, sections were stained with haemotoxylin and eosin (H&E). The histological sections were used to investigate the middle ear inflammation of the mice. Evaluation of mean mucosal thickness was by blinded assessment of a standard 1000 μm length of ME mucosa (avoiding the cochlea and the region close to the Eustachian tube), the mucosal thickness was averaged from five measurements.

To study the lung morphology of *edison* mice, H&E stained sections from adult and perinatal lungs were viewed using a Zeiss Axiostar Plus bright-field microscope and analysed using cell^B^ imaging software (Olympus). The data was analysed as previously described [49].

### Scanning electron microscopy

To study the ultra-structure of the organ of Corti we dissected the inner ears from five 20 wk *Nisch*^*+/+*^ and *Nisch*^*edsn/edsn*^ mice and prepared the samples as previously described [50]. Inner ears were imaged using a JEOL 6010 LV scanning electron microscope under high vacuum conditions.

### Blood and bulla fluid collection

Blood and bulla fluids were collected, as previously described [14], for analysis using Real-time quantitative PCR.

### Real-time quantitative PCR (RT-qPCR)

Real-time quantitative PCR was performed as previously described [14]. For ear fluid analysis, each sample pool comprised the fluid from both ears of four individual samples. For blood analysis each sample pool comprised four individual samples. Murine TaqMan gene expression assays used for analysis were *Hif1a* (Mm01283756_m1), *Il1b* (Mm01336189_m1), *Tnfa* (Mm00443258_m1), *Vegfa* (Mm00437304_m1), *Src* (Mm00436785_m1), *Evi1* (Mm00491303_m1) and *Fbxo11* (Mm01227499_m1). *Ppia* (Mm02342429_g1) was used as the endogenous control.

### Immunohistochemistry

For immunohistochemical analysis, the avidin–biotin complex (ABC) method was used to look for the localization of NISCH, ITGA5, p-PAK, p-LIMK1/2, RAC1, NF-κB p65, FAK and p-SMAD2 in wild-type and mutant mouse ME and lungs. The sections through the ears of mice were de-parafinized, and endogenous peroxidase activity was quenched with 3% hydrogen peroxide in isopropanol for 30 min. Vectastain Elite ABC kit (Vector Laboratories, PK 6101) was used to perform the immunohistochemistry. The antibodies were as follows: rabbit polyclonal anti-NISCH (sc-98980, Santa Cruz Biotechnology), rabbit polyclonal anti-ITGA5 (sc-10729, Santa Cruz Biotechnology), rabbit polyclonal anti-p-αPAK (Thr212) (sc-101772, Santa Cruz Biotechnology), rabbit polyclonal anti-p-LIMK1/2 (Thr508/505) (sc-28409-R, Santa Cruz Biotechnology), rabbit polyclonal anti-RAC1 (sc-95, Santa Cruz Biotechnology), rabbit polyclonal anti-p-SMAD2 (Ser465/467) (AB3849, Chemicon International), rabbit polyclonal anti-FAK (sc-558, Santa Cruz Biotechnology) rabbit polyclonal anti-NF-κB p65 (ab131485, Abcam). The sections were incubated with the antibodies overnight at the following dilutions: p-PAK, 1:50; NISCH, ITGA5, p-LIMK1/2, NF-κB p65, FAK and p-SMAD2, 1:200; Rac1, 1:400. For F4/80 visualisation, sections were treated with 0.05% trypsin in calcium chloride for 20 min at 37°C, blocked with 10% rabbit serum (X0902, DAKO), incubated with rat anti mouse F4/80 (MCA497GA, Serotec) antibody overnight at 1:100 dilution and the next day after the washes were incubated with biotinylated rabbit anti-rat secondary antibody at 1:400 dilution (E0468, DAKO). The serum and the secondary antibody for all the other antibodies were from the Vectastain Elite ABC kit and they were used according to the manufacturer's instructions. DAB+ chromogen system (DAKO K3468) was used to develop the specific signals. The slides were counterstained with haematoxylin.

### Western blot

Total protein extracted from the ME epithelial cells and lungs of two-months-old wild-type, *Nisch*^*edsn/edsn*^ and *Itga5*^*tm1Hyn/+*^; *Nisch*^*edsn/edsn*^ mice were used for the western blot analysis. Each middle ear sample consisted of combined epithelial cells scooped out of both ears of one mouse. Each lung sample consisted of whole lung tissue from one mouse. Either three or four biological replicates were performed for each antibody. The tissues were homogenised in CelLytic MT Cell Lysis Reagent (Sigma C3228), protease inhibitors, phosphatase inhibitors and vanadate and centrifuged at 4°C. Protein concentration was determined using the DC Protein Assay kit (Bio-Rad). Samples (30 μg from the lung samples and 10 μg from the middle ear samples) were loaded into 12% NuPAGE Bis-Tris gel, 7% NuPAGE Tris Acetate gel or 3–8% NuPAGE Tris acetate gels (Invitrogen), blotted onto nitrocellulose membrane (Invitrogen) and immunostained. 5% non-fat milk in TBST was used as blocking solution and antibody diluent. The antibodies and the dilutions they were used at for the western blot analysis were as follows: rabbit polyclonal anti-ITGA5 (sc-10729, Santa Cruz Biotechnology) 1:500, rabbit polyclonal anti-PAK1 (2602, Cell Signaling) 1:1000, rabbit polyclonal anti-p-LIMK1/2 (Thr508/505) (sc-28409-R, Santa Cruz Biotechnology) 1:500, goat polyclonal anti LIMK1 (sc-8387, Santa Cruz Biotechnology) 1:500, rabbit polyclonal anti-RAC1 (sc-95, Santa Cruz Biotechnology) 1:500, rabbit polyclonal anti-FAK (sc-558, Santa Cruz Biotechnology) 1:500, rabbit polyclonal anti-NF-κB p65 (ab131485, Abcam) 1:1000, rabbit polyclonal p-NFκB p65 (Ser 276) (sc-101749, Santa Cruz Biotechnology) 1:500, rabbit polyclonal anti-phospho-FAK (Tyr576) (44-652G, Invitrogen), rabbit polyclonal anti-phospho-SRC (Tyr527) (2105, Cell Signaling), rabbit polyclonal anti-phospho-SMAD2 (Ser465 /467) (3101, Cell Signaling) 1:500 and actin (A 2066, Sigma). Goat anti-rabbit IgG (H+L)-HRP conjugate (1706515, Bio-Rad), 1:3000, was used as a secondary antibody for all the primary antibodies except for LIMK1 for which Rabbit anti-goat IgG (H+L) secondary antibody, HRP, 1:5000 (81–1620, Invitrogen) was used. ECL or ECL 2 (GE Healthcare) were used as detection system.

### Data analysis

All data are given as unadjusted mean +/- SEM (standard error of the mean) unless stated otherwise. Data were analysed to establish normal distribution. Where data was normally distributed an ANOVA or Student’s t-test were conducted. If data was not normally distributed the non-parametric equivalents of these tests were used (Kruskal-Wallis One Way Analysis of Variance on Ranks or Mann-Whitney Rank Sum Test) to establish if data were significant. The Holm-Sidak method (ANOVA) or Dunn’s method (Ranks) was used for multiple comparisons versus a control group. Results with values of *P* < 0.05 were considered statistically significant. SigmaPlot 11.0 software was used to perform all statistical analysis.

## Supporting information

S1 Fig *Nisch*^*edsn/edsn*^ craniofacial defect has no effect on Eustachian tube morphology. **(A)** Characteristic image of a male *Nisch*^*edsn/edsn*^ mutant and a wild-type littermate. **(B)** Weight data for male *edison* mice over a 20 wk longitudinal time course shows *Nisch*^*edsn/edsn*^ mice are significantly smaller than both wild-type and *Nisch*^*edsn/+*^ littermates throughout the time course. In addition, *Nisch*^*edsn/+*^ mice are also smaller than those wild-type for the allele. * *P* < 0.05; ** *P* < 0.01; *** *P* < 0.001. *Nisch*^*+/+*^, n = 9; *Nisch*^*edsn/+*^, n = 20; *Nisch*^*edsn/edsn*^, n = 18 **(C)** Dorsoventral view of a 20 wk wild-type mouse skull showing the measurements used to analyse skull morphology in this study. SL, skull length; SW, skull width; NB, nasal bone; FB, frontal bone; PB, parietal bone. **(D)** Allometric comparisons against skull length show abnormal growth in *Nisch*^*edsn/edsn*^ skulls at the nasal bone and frontal bone. ns *P* > 0.05; * *P* < 0.05; *** *P* < 0.001. *Nisch*^*+/+*^, n = 13; *Nisch*^*edsn/+*^, n = 18; *Nisch*^*edsn/edsn*^, n = 12. **(E)** Dissected and stained skulls of *Nisch*^*+/+*^, *Nisch*^*edsn/+*^ and *Nisch*^*edsn/edsn*^ 20 wk mice. Eustachian tube (ET) angle measurements are indicated by dashed lines. R, right ear; L, left ear. **(F)** Mean angle between the midline of the skull and the bony part of the left and the right ET. ns *P* > 0.05. *Nisch*^*+/+*^, n = 6; *Nisch*^*edsn/+*^, n = 6; *Nisch*^*edsn/edsn*^, n = 6. Error bars indicate standard error of mean. Statistics were conducted using one-way ANOVA’s and Holm-Sidak’s multiple comparison tests for post-hoc analysis.
(TIF)

S2 Fig No additional auditory abnormalities contributing to *Nisch*^*edsn/edsn*^ hearing deficit. **(A)** Frequency-specific ABR thresholds (8 kHz, 16 kHz, 32 kHz and click-evoked) of *Nisch*^*edsn/edsn*^ mice across a longitudinal time course displayed parallel shifts in audiometric profiles across frequencies, consistent with a conductive hearing loss. n = 5. **(B)** H&E mid-modiolar sections of the cochlea showed comparable structure of inner ears in 20 wk *Nisch*^*+/+*^ and *Nisch*^*edsn/edsn*^ mice. *Nisch*^*+/+*^ n = 5; *Nisch*^*edsn/edsn*^ n = 5. Scale bar = 1 mm. OC, organ of Corti; SG, spiral ganglion; SL, spiral ligament; RM, Reissner’s membrane. **(C)** H&E sections of the organ of Corti from the mid cochlear turn displayed no differences in the morphology between *Nisch*^*+/+*^ and *Nisch*^*edsn/edsn*^ mice at 20 wk. *Nisch*^*+/+*^ n = 5; *Nisch*^*edsn/edsn*^ n = 5. Scale bar = 100 μm. OHC, outer hair cell; IHC, inner hair cell. **(D)** Scanning electron microscopy (SEM) images showed normal hair cell morphology in 20 wk *Nisch*^*+/+*^ and *Nisch*^*edsn/edsn*^ mice. *Nisch*^*+/+*^ n = 5; *Nisch*^*edsn/edsn*^ n = 5. Scale bar = 10 μm.
(TIF)

S3 Fig Gene expression in *Nisch*^*edsn/edsn*^ bulla fluid inflammatory cells compared with white blood cells. Relative Quantification (RQ) of gene expression using TaqMan RT-qPCR for *Nisch*^*edsn/edsn*^ mice at 20 wk. Blood, n = 3 pools; Ear fluid, n = 3 pools. ns *P* > 0.05; ** *P* < 0.01; *** *P* < 0.001. Error bars indicate 95% confidence interval. A Student's t-test of the replicate 2^(−ΔCt)^ values was performed to analyse the data.
(TIF)

S4 Fig *Itga5*^*tm1Hyn/+*^ *Nisch*^*edsn/+*^ mice develop a mild late-onset otitis media. **(A, B)** Visual inspection of the tympanic membrane was used as a semi-quantitative measure for the prevalence of OM. (A) *Itga5*^*tm1Hyn/+*^; *Nisch*^*+/+*^ mice show a small incidence of unilateral OM at 4 wk, whereas in (B) *Itga5*^*tm1Hyn/+*^; *Nisch*^*edsn/+*^ animals prevalence of OM increases with age compared to littermates with onset at 12 wk. **(C-F)** H&E stained transverse sections of the MEC and mucoperiosteum, in 20 wk (C, E) *Itga5*^*tm1Hyn/+*^; *Nisch*^*+/+*^ and (D, F) *Itga5*^*tm1Hyn/+*^; *Nisch*^*edsn/+*^ animals. *Itga5*^*tm1Hyn/+*^; *Nisch*^*edsn/+*^ mice develop OM with a diffuse mucosal inflammation of mild severity, with the presence of a cellular middle ear effusion. C, cochlea; ET, Eustachian tube; E, exudate; MEC, middle ear cavity; MP, mucoperiosteum (arrowheads); TB, temporal bone; TM, tympanic membrane. C, D scale bar = 2 mm; E, F scale bar = 200 μm.
(TIF)

S5 Fig Protein expression analysis of NISCH interacting partners and downstream pathways in lung. Immunohistochemistry of lung sections of *Nisch*^*+/+*^, *Nisch*^*edsn/edsn*^ and *Itga5*^*tm1Hyn/+*^; *Nisch*^*edsn/edsn*^ mice at 3 wk and total lung extracts at 8 wk, with **(A)** NISCH, **(B)** ITGA5, **(C)** PAK1, **(D)** p-LIMK1/2, **(E)** RAC1, **(F)** NF-κB p65, **(G)** p-SMAD2 and **(H)** FAK antibodies. To quantify the results from the staining, airway epithelial cells in wild-types and mutants were counted in three different regions from four lungs for each genotype. The results presented from the western blots are from four independent experiments for all the antibodies except for p-LIMK1/2, where three independent experiments were used to present the data. Scale bar = 100 μm. * *P* < 0.05. Error bars indicate standard error of mean. Immunohistochemistry data was analysed by one-way ANOVAs and Holm-Sidak’s multiple comparison procedures for post-hoc testing. For western blot analysis a Student’s t-test was performed.
(TIF)
