# Supplementary figures and images for: A mutation in Nischarin causes otitis media via LIMK1 and NF-κB pathways
Source: PLoS Genet. 2017 Aug 14;13(8):e1006969. doi: 10.1371/journal.pgen.1006969 (PMC5570507; doi:10.1371/journal.pgen.1006969)

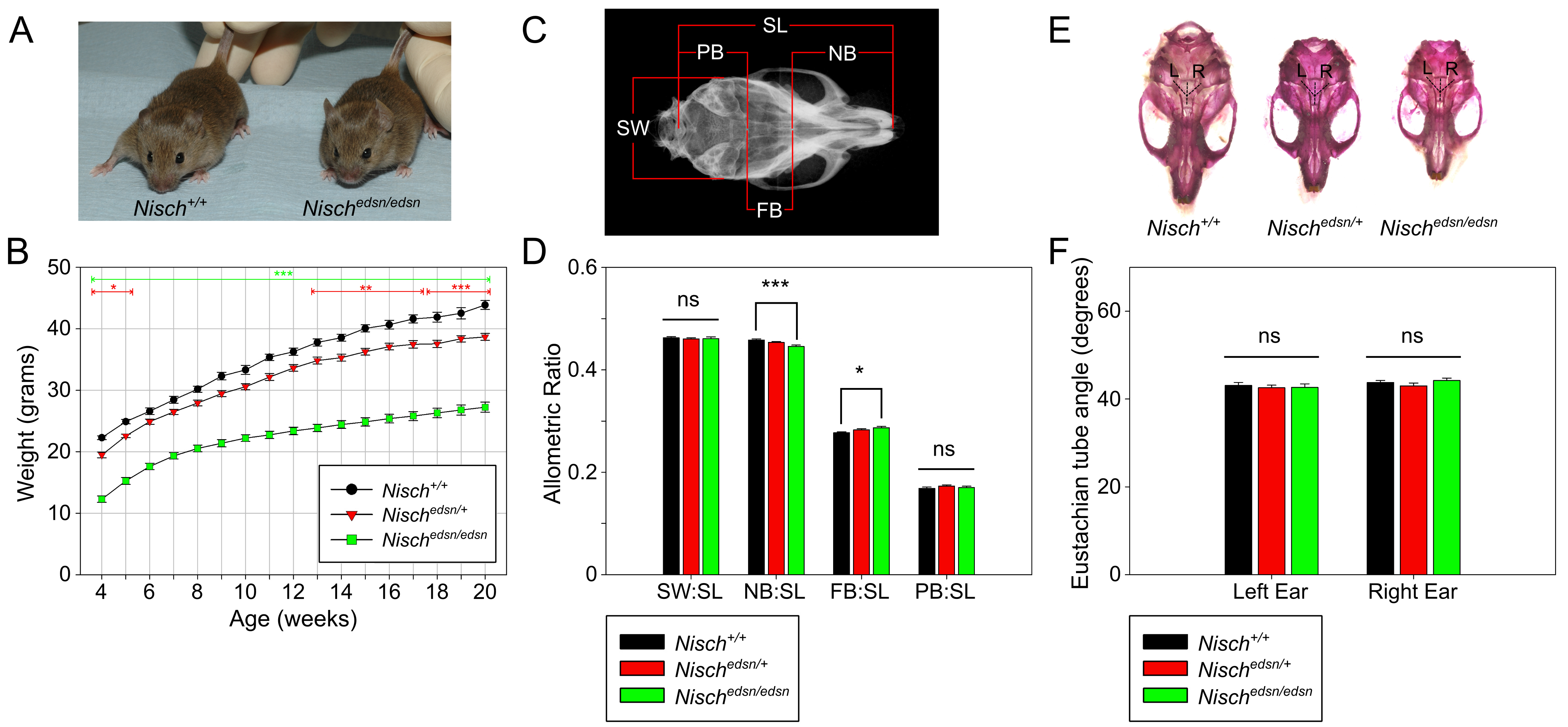

Supplement: S1 Fig — (A) Characteristic image of a male Nischedsn/edsn mutant and a wild-type littermate. (B) Weight data for male edison mice over a 20 wk longitudinal time course shows Nischedsn/edsn mice are significantly smaller than both wild-type and Nischedsn/+ littermates throughout the time course. In addition, Nischedsn/+ mice are also smaller than those wild-type for the allele. * P < 0.05; ** P < 0.01; *** P < 0.001. Nisch+/+, n = 9; Nischedsn/+, n = 20; Nischedsn/edsn, n = 18 (C) Dorsoventral view of a 20 wk wild-type mouse skull showing the measurements used to analyse skull morphology in this study. SL, skull length; SW, skull width; NB, nasal bone; FB, frontal bone; PB, parietal bone. (D) Allometric comparisons against skull length show abnormal growth in Nischedsn/edsn skulls at the nasal bone and frontal bone. ns P > 0.05; * P < 0.05; *** P < 0.001. Nisch+/+, n = 13; Nischedsn/+, n = 18; Nischedsn/edsn, n = 12. (E) Dissected and stained skulls of Nisch+/+, Nischedsn/+ and Nischedsn/edsn 20 wk mice. Eustachian tube (ET) angle measurements are indicated by dashed lines. R, right ear; L, left ear. (F) Mean angle between the midline of the skull and the bony part of the left and the right ET. ns P > 0.05. Nisch+/+, n = 6; Nischedsn/+, n = 6; Nischedsn/edsn, n = 6. Error bars indicate standard error of mean. Statistics were conducted using one-way ANOVA’s and Holm-Sidak’s multiple comparison tests for post-hoc analysis. (TIF) [file pgen.1006969.s001.tif]

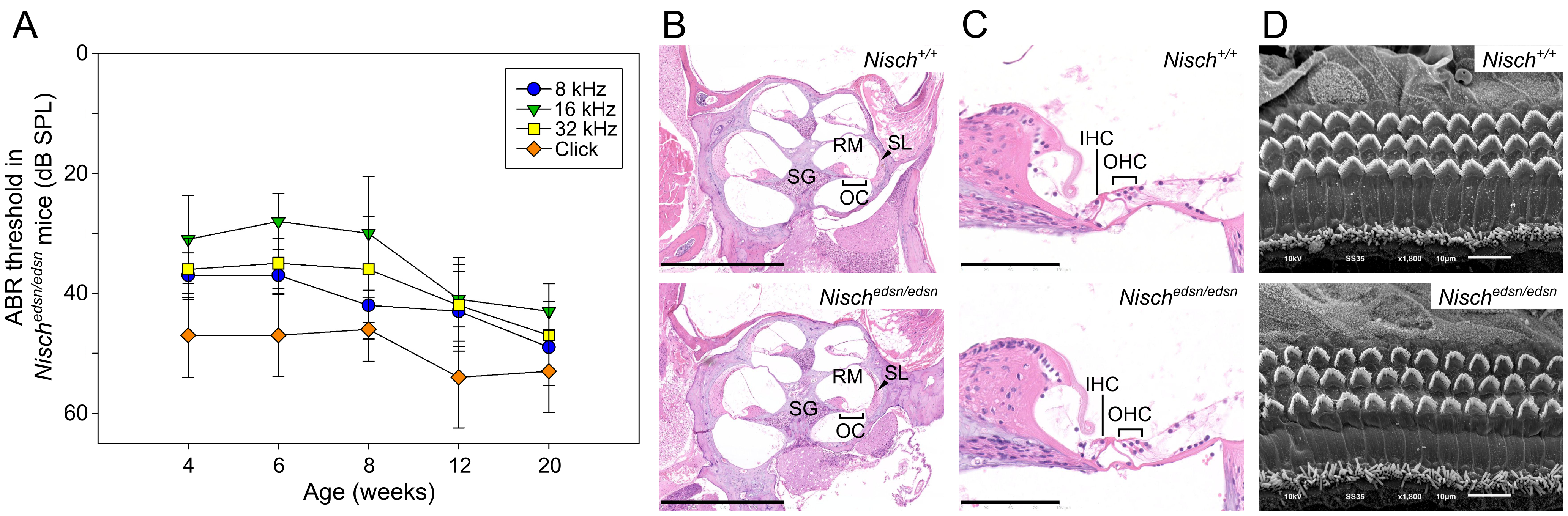

Supplement: S2 Fig — (A) Frequency-specific ABR thresholds (8 kHz, 16 kHz, 32 kHz and click-evoked) of Nischedsn/edsn mice across a longitudinal time course displayed parallel shifts in audiometric profiles across frequencies, consistent with a conductive hearing loss. n = 5. (B) H&E mid-modiolar sections of the cochlea showed comparable structure of inner ears in 20 wk Nisch+/+ and Nischedsn/edsn mice. Nisch+/+ n = 5; Nischedsn/edsn n = 5. Scale bar = 1 mm. OC, organ of Corti; SG, spiral ganglion; SL, spiral ligament; RM, Reissner’s membrane. (C) H&E sections of the organ of Corti from the mid cochlear turn displayed no differences in the morphology between Nisch+/+ and Nischedsn/edsn mice at 20 wk. Nisch+/+ n = 5; Nischedsn/edsn n = 5. Scale bar = 100 μm. OHC, outer hair cell; IHC, inner hair cell. (D) Scanning electron microscopy (SEM) images showed normal hair cell morphology in 20 wk Nisch+/+ and Nischedsn/edsn mice. Nisch+/+ n = 5; Nischedsn/edsn n = 5. Scale bar = 10 μm. (TIF) [file pgen.1006969.s002.tif]

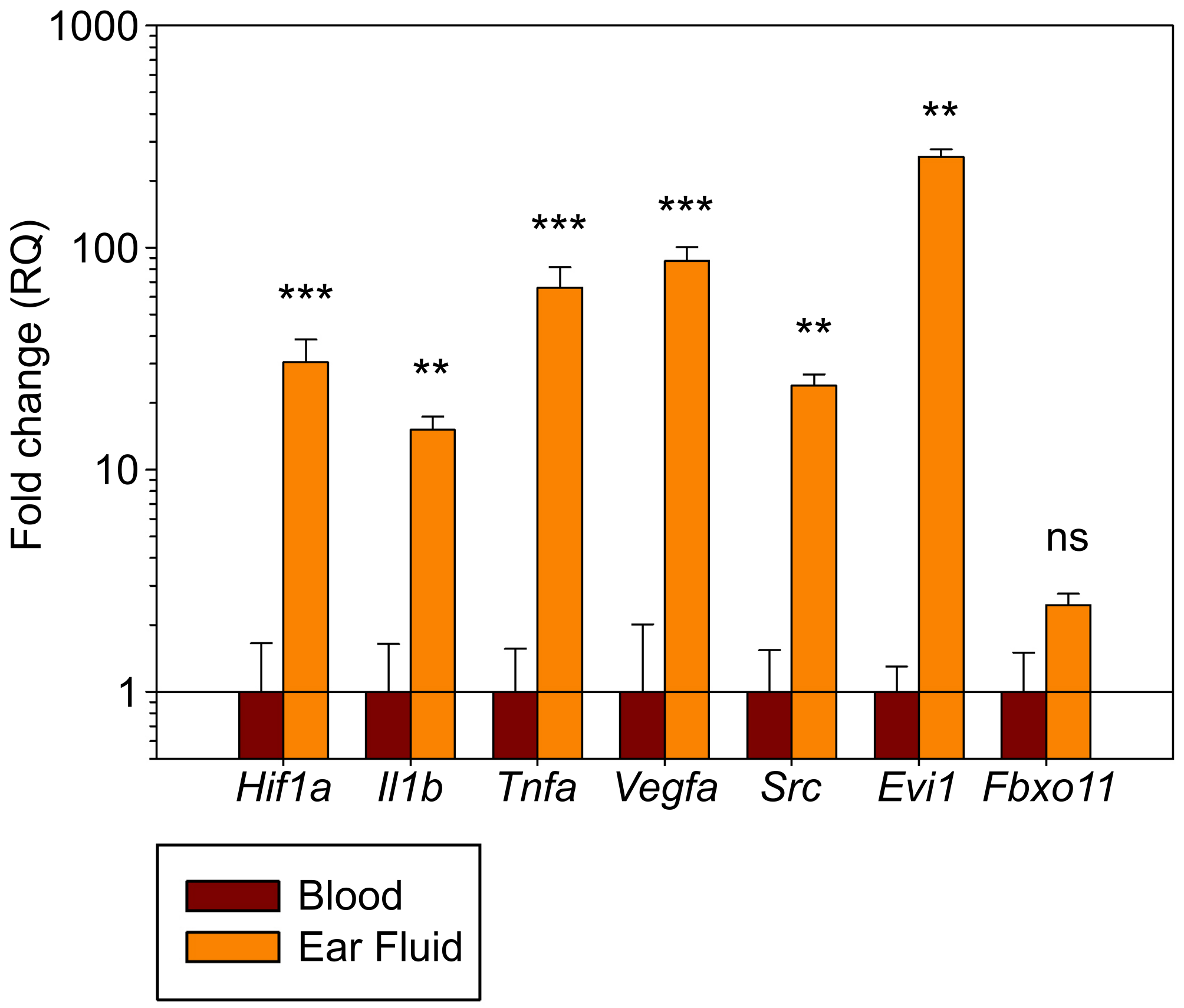

Supplement: S3 Fig — Relative Quantification (RQ) of gene expression using TaqMan RT-qPCR for Nischedsn/edsn mice at 20 wk. Blood, n = 3 pools; Ear fluid, n = 3 pools. ns P > 0.05; ** P < 0.01; *** P < 0.001. Error bars indicate 95% confidence interval. A Student's t-test of the replicate 2(−ΔCt) values was performed to analyse the data. (TIF) [file pgen.1006969.s003.tif]

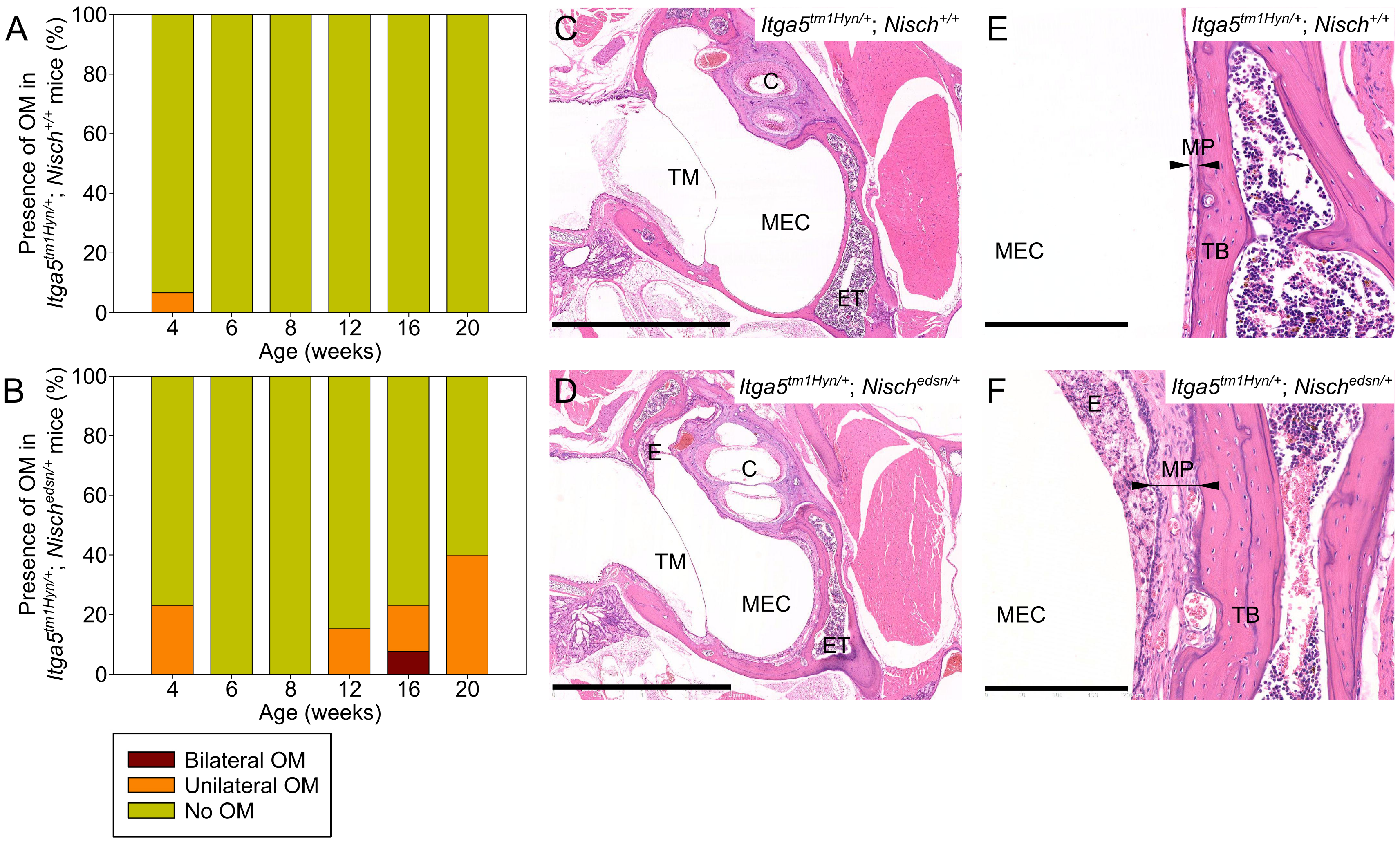

Supplement: S4 Fig — (A, B) Visual inspection of the tympanic membrane was used as a semi-quantitative measure for the prevalence of OM. (A) Itga5tm1Hyn/+; Nisch+/+ mice show a small incidence of unilateral OM at 4 wk, whereas in (B) Itga5tm1Hyn/+; Nischedsn/+ animals prevalence of OM increases with age compared to littermates with onset at 12 wk. (C-F) H&E stained transverse sections of the MEC and mucoperiosteum, in 20 wk (C, E) Itga5tm1Hyn/+; Nisch+/+ and (D, F) Itga5tm1Hyn/+; Nischedsn/+ animals. Itga5tm1Hyn/+; Nischedsn/+ mice develop OM with a diffuse mucosal inflammation of mild severity, with the presence of a cellular middle ear effusion. C, cochlea; ET, Eustachian tube; E, exudate; MEC, middle ear cavity; MP, mucoperiosteum (arrowheads); TB, temporal bone; TM, tympanic membrane. C, D scale bar = 2 mm; E, F scale bar = 200 μm. (TIF) [file pgen.1006969.s004.tif]

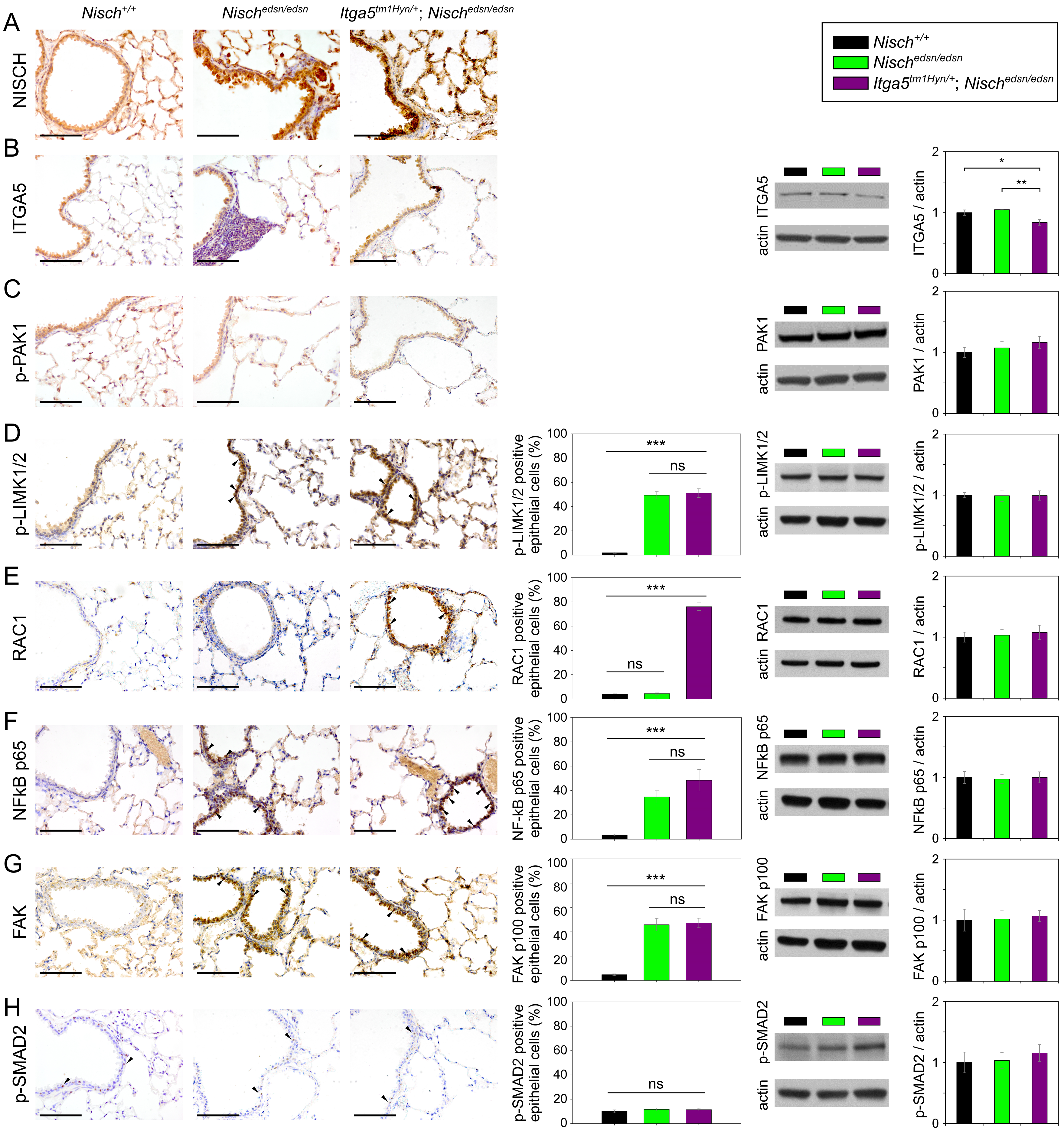

Supplement: S5 Fig — Immunohistochemistry of lung sections of Nisch+/+, Nischedsn/edsn and Itga5tm1Hyn/+; Nischedsn/edsn mice at 3 wk and total lung extracts at 8 wk, with (A) NISCH, (B) ITGA5, (C) PAK1, (D) p-LIMK1/2, (E) RAC1, (F) NF-κB p65, (G) p-SMAD2 and (H) FAK antibodies. To quantify the results from the staining, airway epithelial cells in wild-types and mutants were counted in three different regions from four lungs for each genotype. The results presented from the western blots are from four independent experiments for all the antibodies except for p-LIMK1/2, where three independent experiments were used to present the data. Scale bar = 100 μm. * P < 0.05. Error bars indicate standard error of mean. Immunohistochemistry data was analysed by one-way ANOVAs and Holm-Sidak’s multiple comparison procedures for post-hoc testing. For western blot analysis a Student’s t-test was performed. (TIF) [file pgen.1006969.s005.tif]
